# Mesenchymal Stem Cells Beyond Regenerative Medicine

**DOI:** 10.3389/fcell.2020.00072

**Published:** 2020-02-18

**Authors:** Riam Shammaa, Abed El-Hakim El-Kadiry, Jamilah Abusarah, Moutih Rafei

**Affiliations:** ^1^Canadian Centre for Regenerative Therapy, Toronto, ON, Canada; ^2^IntelliStem Technologies Inc., Toronto, ON, Canada; ^3^Department of Family and Community Medicine, University of Toronto, Toronto, ON, Canada; ^4^Laboratory of Thrombosis and Hemostasis, Montreal Heart Institute, Montreal, QC, Canada; ^5^Department of Pharmacology and Physiology, Université de Montréal, Montreal, QC, Canada; ^6^Department of Microbiology and Immunology, McGill University, Montreal, QC, Canada; ^7^Department of Microbiology, Infectious Diseases and Immunology, Université de Montréal, Montreal, QC, Canada; ^8^Molecular Biology Program, Université de Montréal, Montreal, QC, Canada

**Keywords:** MSC, regeneration, autoimmunity, cancer, antigen, vaccine

## Abstract

Mesenchymal stem cells (MSCs) are competent suitors of cellular therapy due to their therapeutic impact on tissue degeneration and immune-based pathologies. Additionally, their homing and immunomodulatory properties can be exploited in cancer malignancies to transport pharmacological entities, produce anti-neoplastic agents, or induce anti-tumor immunity. Herein, we create a portfolio for MSC properties, showcasing their distinct multiple therapeutic utilities and successes/challenges thereof in both animal studies and clinical trials. We further highlight the promising potential of MSCs not only in cancer management but also in instigating tumor-specific immunity – i.e., cancer vaccination. Finally, we reflect on the possible reasons impeding the clinical advancement of MSC-based cancer vaccines to assist in contriving novel methodologies from which a therapeutic milestone might emanate.

## Introduction

Broadly distributed among tissues, MSCs are first generation adult stem cells of mesodermal non-hematopoietic origins. They were originally reported in bone marrow (BM) by Friedenstein et al. (1968, 1970) and later identified in adipose tissue, peripheral blood, cruciate ligaments, dental pulp, menses blood, amniotic fluid, fallopian tube, placenta, umbilical cord, and endometrial polyps (Caplan, 1991; Bianco et al., 2008; Ding et al., 2011; Sheng, 2015; Ullah et al., 2015). According to the International Society for Cellular Therapy (ISCT), MSCs are characterized by their (i) adherence to plastic, (ii) cell surface expression of CD73, CD90, and CD105 but not CD45, CD34, CD14, CD11b, CD79α, CD19, and HLA-DR (hematopoietic cell markers), and (iii) multipotency, the ability to differentiate into various mesodermal cell lineages such as osteoblasts, chondroblasts, and adipocytes (Dominici et al., 2006). However, the ISCT definition is no longer standardized as MSC identification criteria continue to change. Exemplifying this are the discovery that MSCs can also differentiate into cells of ectodermal and endodermal parentage (Wei et al., 2013) and the inclusion of novel surface markers to their identity (CD165, CD276, and CD82) (Al-Nbaheen et al., 2013). Several studies on MSC lineages have also identified distinctive molecular (Al-Nbaheen et al., 2013; Ullah et al., 2015; Wu et al., 2018), proliferation/differentiation (Kern et al., 2006), and functional properties (Keyser et al., 2007), accrediting the fact that their biology is still partially intelligible. The conventional notion, however, is that MSCs are (i) genomically stable, (ii) highly accessible, (iii) easy to isolate and expand, (iv) immune-privileged (low expression of MHC I/II and co-stimulatory molecules and – further explained in Section “Immunological Properties: A Paradigm” – immunomodulation), and – unlike other types of stem cells – (v) non-teratogenic and ethically conforming (Wei et al., 2013). Additionally, a number of reports showing that BM-MSCs from healthy donors perform better in proliferation/differentiation and secretion criteria compared to BM-MSCs from osteoarthritic (Murphy et al., 2002) and Gaucher disease patients (Campeau et al., 2009) corroborate that MSCs play a physiological role in homeostatic tissue maintenance, whereas their disturbance may foster disease pathogenesis. In this review article, we recapitulate a vast literature on MSC assets, demonstrating from preclinical and clinical perspectives how they render them fit candidates for cellular therapy. Finally, we discuss the trend of MSC utility against tumors to bridge to the highlight of this review – MSCs as cancer vaccines – pinpointing the flaws halting their clinical effectiveness while offering novel insight on how to overcome them.

## Msc Fitness for Cellular Therapy

### Regenerative Properties

Numerous studies illustrate the regenerative potential of MSCs based on their homing, engraftment, (trans)differentiation, and ability to replace apoptotic/necrotic tissue or dissipate paracrine signaling to boost injured tissue function (Prockop, 1997). *In vitro*-cultured systemically-infused MSCs home *via* their chemokine and toll-like receptors (TLRs) into several organs including BM, heart, and liver in which they can persist for prolonged periods of time (Devine et al., 2001; Allers et al., 2004; Lüttichau et al., 2005; Tomchuck et al., 2008). Factors in favor of homing are young recipient age, irradiation, decreased cell passage number, cytokines/inflammation, as well as increased chemokine receptor and TLR expression (Horwitz et al., 2002; François et al., 2006; Shi et al., 2007; Kyriakou et al., 2008; Tomchuck et al., 2008). Besides the former receptors, MSCs express a variety of adhesion molecules, endopeptidases, and growth factors in addition to their cognate receptors, which facilitate MSC tethering, endothelial rolling, and transmigration to tissues (De Becker and Van Riet, 2016). MSCs might mobilize as well under several stimuli such as growth factors (Asahara et al., 1999) and xenobiotics (Llevadot et al., 2001) before engrafting into tissues where they either (trans)differentiate to the constituent cells (Prockop et al., 2010) or secrete various humoral factors in the extracellular space such as cytokines, chemokines, and mRNA/microRNA (miRNA)-containing microvesicles to modulate tissue function (Wei et al., 2013). Factors influencing tissue engraftment efficiency are cell death, immune rejection, and first-pass lung entrapment which can be overcome by optimizing delivery methods, ameliorating target tissue receptivity, and schooling MSCs to resist tissue hostility (Kean et al., 2013; Ezquer et al., 2017).

Following adherence to plastic *in vitro* or tissue engraftment *in vivo*, MSCs form colonies and (trans)differentiate into a myriad of cell lineages (Kuznetsov et al., 1997; Li H. et al., 2006; Wang et al., 2012; Vonk et al., 2018). For this to occur, their microenvironment must contain multiple mitogenic or stimulating factors (Tontonoz et al., 1994; Sekiya et al., 2002; Lucarelli et al., 2003; Solchaga et al., 2005; Fontaine et al., 2008; Inada et al., 2008; Pavlova et al., 2012); be subjected to hypoxic conditions (Mohyeldin et al., 2010; Zhang et al., 2019); or scaffolded to closely mimic organ architecture or function (Ouyang et al., 2003; Ohgushi et al., 2005). However, a newer understanding of the regenerative abilities of MSCs *in vivo* later emerged, linking tissue regrowth not to MSC (trans)differentiation exclusively but rather to autocrine and paracrine signaling transduced through their communication with local stimuli (Crisostomo et al., 2008), growth factors (Hahn et al., 2008), and inflammatory mediators (Haynesworth et al., 1996). This creates a rich nutritive milieu to which cells in the vicinity also contribute (Caplan and Dennis, 2006). Within the trophic environment are factors dictating angiogenesis (Min et al., 2002), hindrance of apoptosis (Xu et al., 2007), inhibition of fibrosis, mitosis in local tissue (Takahashi et al., 1999), and formation of a structural niche with other resident stem cells (Méndez-Ferrer et al., 2010). In addition, MSCs secrete microvesicles and exosomes which contain pro-angiogenic growth factors and miRNA as a means to establish cell-to-cell communication (Gong et al., 2017; Phinney and Pittenger, 2017). On the other hand, multiple factors can still hamper MSC regenerative functions such as temperature, media type (Kubrova et al., 2019), interference of plastic adherence with cellular function (Mabuchi et al., 2012), chromosomal abnormalities, transformation, and tumor growth especially in MSCs of murine sources. Having said that, isolation and culture protocols recently developed for human MSCs derived from healthy subjects appear as promising endeavors to overcome those hurdles (Bernardo et al., 2007; Law and Chaudhuri, 2013; Conforti et al., 2016). For example, transformation and persistence were addressed in a protocol that uses skin tissue of patients undergoing any relevant medical intervention. To obtain MSCs, the tissues are disinfected and enzymatically digested in good manufacturing practice (GMP). Cell yields are then sorted with antibody-coupled magnetic beads, and cultured MSCs are validated according to ISCT criteria. Finally, several tests are performed to assess *in vivo* toxicity, tumorigenicity, and biodistribution/persistence (Tappenbeck et al., 2019). The data of another clinical study, which warranted its authors an “orphan designation” in Germany for graft-versus-host disease (GvHD) treatment using MSCs, authenticate the effectiveness of such protocol. Indeed, generating the MSCs entailed the enrichment of BM aspirates of several donors using an automated cell separation unit and processing system followed by the expansion of MSCs in culture over 14 days. From this bank, clinical-grade MSCs are obtained and cultured in platelet lysate serum-free media whose utility eliminates the risks associated with the use of fetal bovine serum such as immunogenicity and pathogenicity (Kuçi et al., 2016; Bader et al., 2018).

### Immunological Properties: A Paradigm

In addition to its tissue repair characteristics, the secretome of MSCs displays immunomodulatory properties. This is evident in the ability of MSCs to interfere with the cell cycle (G0/G1 phase arrest), hinder the responses of naïve and memory T cells, inhibit the activation and proliferation of effector T cells, and induce regulatory T cell (T_*reg*_) function (Krampera et al., 2003; Siegel et al., 2009; Duffy et al., 2011; Haddad and Saldanha-Araujo, 2014). Such immunosuppressive activity essentially ensues in response to inflammatory signals including interferon-γ (IFN-γ), TNF-α, and interleukin-1 (IL-1). These pro-inflammatory molecules prime MSCs, such that they induce the secretion of multiple soluble immunosuppressive molecules and the upregulation of inhibitory surface co-receptors including programed death-ligand 1 (PD-L1) (Sheng et al., 2008). Those mechanisms are protective against immune cells such as natural killer (NK) cells which become cytolytic upon activation by inflammatory signals, the same signals inducing the upregulation of MHC class I/II on MSCs and subsequently their susceptibility to NK cell cytotoxicity. Interestingly, NK cells/MSCs ratio is the determinant of the inhibitory power balance. For example, lower ratios tip the suppressive balance in favor of MSCs which become capable of inducing phenotypic and secretory changes in NK cells *via* physical and paracrine interactions, thereby restricting their cytotoxicity and proliferation (Sotiropoulou et al., 2006; Jewett et al., 2010; Spaggiari and Moretta, 2012). Pro-inflammatory signals also support MSC differentiation through multiple receptors like TLRs and signaling pathways like NF-κB, p38 mitogen-activated protein (MAP) kinase, and β-catenin, ultimately inducing the transcription of lineage-specific genes (Cheng et al., 2008; Wei et al., 2013; Chen et al., 2016; Liu et al., 2018). For instance, NF-κB and MAP kinase pathways are activated by stromal cell-derived factor-1 (SDF-1), a pleotropic chemokine secreted by several cells and organs, which acts as a chemoattractant for MSCs in regenerative settings (Kucia et al., 2004). Elsewhere, however, NF-κB upregulation by pro-inflammatory cytokines was negatively correlated with MSC differentiation, particularly osteogenesis (Ansari et al., 2017). In contrast, the absence of strong inflammatory stimuli (e.g., low levels of inflammatory or anti-inflammatory cytokines) does not trigger the production of immunosuppressive factors, thus permitting a pro-inflammatory environment to takeover. This is evident in a few studies showing that *in vivo* transplantation of unchallenged allogeneic MSCs evokes cellular and humoral immune responses (Eliopoulos and Galipeau, 2002; Poncelet et al., 2007; Renner et al., 2009). Furthermore, inflammatory signals allow MSCs to govern the activity of multiple innate and adaptive immune cells including B cells, neutrophils, and macrophages through secreted soluble factors such as prostaglandins, chemokine ligands, interleukins (ILs), growth factors, and nitric oxide (NO) (Singer and Caplan, 2011). Those factors interfere with inflammatory signaling pathways (e.g., STAT3), ultimately mitigating antigen presentation and humoral immunity (Rafei et al., 2008; Loebinger and Janes, 2010). In addition to their secretome, MSCs can mitigate mixed lymphocyte reactions by physically hindering the contact of T cells with antigen presenting cells (APCs) (Krampera et al., 2003); JAG1-NOTCH interaction is shown to partake in the process (Liotta et al., 2008). Overall, immunosuppressive MSCs, later designated as MSC2, contribute to tissue healing and regeneration not only by impeding injury-driven autoimmune responses but also by educating macrophages, *via* IL-6, toward a proangiogenic M2 phenotype. M2 macrophages, therefore, tip the balance of T-cell responses in favor of immune regulation (anti-inflammatory T_*regs*_) (Eggenhofer and Hoogduijn, 2012; Bernardo and Fibbe, 2013; Chung and Son, 2014).

Paradoxically, few reports have challenged the sole immunosuppressive dogma, offering a novel insight into the polarization of MSCs toward another “pro-inflammatory” type, in a similar fashion to “macrophage polarization” (Krampera, 2011). Waterman et al. designated this pro-inflammatory phenotype MSC1. Consequently, MSC2 identified its immunosuppressive counterpart (Waterman et al., 2010). The polarization into either phenotype is originally induced by TLRs and is ligand-specific. For instance, TLR3 and TLR4 priming by, respectively, poly(I:C) and lipopolysaccharide induce the MSC1 phenotype. In the process, downstream TLR signaling instigates pro-inflammatory secretome patterns (ILs, chemokine ligands, growth factors, apoptosis-inducing ligands) and impairs JAG1-NOTCH interaction between MSCs and T cells. This prevents MSC-mediated immunosuppression (Liotta et al., 2008; Romieu-Mourez et al., 2009) and permits IFN-γ-driven MSC antigen presentation to CD4^+^ and CD8^+^ T cells, thereby evoking immune activation (Chan et al., 2006; François et al., 2009). Similar observations are evident in co-cultures of MSCs and B cells, where the latter’s proliferation, cytokine expression, and differentiation are improved (Rasmusson et al., 2007). On the other hand, immunosuppressive secretome patterns (IDO, prostaglandins) ensue downstream TLR signaling during MSC2 polarization (Waterman et al., 2010). Plus, MSC polarization is TLR type-specific. For instance, TLR4-primed MSCs polarize into MSC1, while TLR3 priming favors the immunosuppressive MSC2 profile in certain studies (Waterman et al., 2010) and MSC1 in others (Romieu-Mourez et al., 2009; Kota et al., 2014). Besides differences in TLR-ligand interactions and TLR type signaling, factors such as ligand concentrations (low concentrations license MSC1 phenotype), priming duration, microenvironment (cytokines, growth factors, stimulants), infections/diseases, tissue lesions, and MSC-T cell engagement timing are also at play in polarization licensing (Krampera, 2011; Strioga et al., 2012).

Despite its controversy, MSC polarization is thought to be part of tissue maintenance, where both distinct phenotypes homogeneously act in injury settings. To this extent, MSC1 may be important early in the process to drive chemotaxis and subsequent reparative processes, while MSC2 may act later to resolve inflammatory tissue injury (Romieu-Mourez et al., 2009; Waterman et al., 2010). Similarly, the process can be exploited not only in regenerative medicine, which depends on inflammatory signals but also in cancer management which, as later discussed, depends on MSC inflammatory and migratory properties, both of which are induced by TLR priming (Waterman et al., 2010).

## MSCs in Therapy: Achievements and Pitfalls

### Regenerative Medicine

The regenerative and immunological assets of MSCs (see Sections “Regenerative Properties” and “Immunological Properties: A Paradigm”) are widely exploited in degenerative settings. In animal models of myocardial infarction (MI), percutaneously injected allogeneic MSCs ameliorated ventricular fibrosis and scarring. Reduced infarct size, myocardial regeneration, enhanced cardiac metabolism and hemodynamics were also recorded (Amado et al., 2005; Cai et al., 2016). In *E. coli* endotoxin-injured human lungs, administration of allogeneic human MSCs reduced extravascular fluid and septal thickening, enhanced alveolar fluid transport, and restored the fluid balance of alveolar compartments (Lee et al., 2009). In rat models of retinal degeneration, the injection of MSCs into the subretinal space enhanced the viability of photoreceptor cells without replacing them (Inoue et al., 2007). In various mouse models of excisional wound healing, the application of MSC-conditioned media enriched in chemokines and cytokines increased the infiltration of macrophages and endothelial progenitor cells into the wounded area (Wu et al., 2007; Chen L. et al., 2008; Sasaki et al., 2008). Similar repair mechanisms induced by MSCs were described in the context of corneal injury (Roddy et al., 2011), colitis (Hayashi et al., 2008), neurodegenerative disorders (Tsai et al., 2019), hepatic injury (Anger et al., 2019), cardiac hypertrophy (Cai et al., 2015), and acute renal failure (Tögel et al., 2005).

A more sophisticated approach in regenerative medicine is MSC engineering on both genetic and architectural levels. In the former, MSC gene expression is altered through viral vector- or electroporation-mediated gene transfer; then their homing capacity to injured/ischemic sites is utilized for local delivery of overexpressed therapeutic genes. Examples on MSC-delivered genes are SDF-1 to ameliorate MI and ischemic brain injury (Penn and Khalil, 2008), glucagon-like peptide-1 to reduce amyloid deposition in Alzheimer’s brains (Klinge et al., 2011), and IL-10 to restrain collagen-induced arthritis (Choi et al., 2008). Architectural MSC engineering involves cell culturing to obtain cellular sheets which can be further maintained in organ-specific stimulating media or assembled onto organ scaffolds to restore injured or defective tissue [e.g., bone regeneration (Yorukoglu et al., 2017) and spinal cord injury (Zeng et al., 2011) applications].

This preclinical success permitted the transit to human studies, with no records of toxicity or tumorigenicity with the use of GMP-compliant human MSCs suitable for clinical trial use (Tappenbeck et al., 2019). Up to this date, 921 clinical studies employing MSCs as the primary intervention have been registered, 704 of which date between 2011 and 2019 (U. S. National Library of Medicine, 2019). This booming, particularly in the last decade of the current century, is indicative of MSC potential to ameliorate a plethora of degenerative diseases (further elaborated in Table 1) (Wei et al., 2013), bearing simultaneously their major implication in physiological tissue maintenance (Murphy et al., 2002; Campeau et al., 2009).

**TABLE 1:** Clinical outcomes of MSC utility in regenerative therapy.

**Clinical condition**	**Regenerative outcomes**	**References/NCT**
Osteogenesis imperfecta	- Improvement of bone growth - Alleviation of fracture	Horwitz et al., 1999
Crohn’s disease	Coverage of fistula	NCT01157650 (García-Olmo et al., 2005)
Deep thermal skin burns	- Restoration of wounds - Trigger of neoangiogenesis	Rasulov et al., 2005
Periodontal defects	- Reduction of pocket depth - Suppression of bleeding - Amelioration of teeth mobility	Yamada et al., 2006
Drug-resistant pulmonary tuberculosis	- Halting bacterial discharge - Resolution of tissular cavity	Erokhin et al., 2008
Liver cirrhosis	Amelioration of liver injury	NCT00420134 (Kharaziha et al., 2009) NCT00956891 (Peng et al., 2011)
Diabetic foot	Enhancement of perfusion	Lu et al., 2011
Chondral defects	- Pain alleviation - Increased activity scores - Improved histological façades	Kyriakidis et al., 2019
Maxillofacial bone defects	Increased bone cyst density	NCT01389661 (Redondo et al., 2018)

Nevertheless, the clinical utility of MSCs faces various limitations including cell source availability (De Bari et al., 2001; Fitzsimmons et al., 2018) and specificity (De Ugarte et al., 2003; Sudres et al., 2006), clinical-grade production compliance with GMP (Sensebé, 2008), scalability (Fitzsimmons et al., 2018), administration timing (Tisato et al., 2007; Polchert et al., 2008; Le Blanc et al., 2008) and technique (Singh et al., 2016), engraftment rate (Fouillard et al., 2003; Le Blanc et al., 2008), polarization control (Polchert et al., 2008; Waterman et al., 2010), localization post-transplant (Law and Chaudhuri, 2013), and tissue persistence (Togel et al., 2005). This is explanatory of the limited number of MSC-based final stage trials and approved biopharmaceutical products. Until 2019, 50 studies have hit Phase III, with only 14 completed (NIH, 2019). Therefrom, 11 MSC-based therapies emanated (BioInformant, 2019) for the treatment of 7 degenerative and immune-based conditions including knee cartilage defects, hip joint avascular necrosis, and coronary angioplasty-reperfused acute MI (PHARMICELL, 2011; ANTEROGEN, 2012; Corestem, 2015; European Medicines Agency, 2017; MilliporeSigma, 2017; Orthocell, 2017; European Medicines Agency, 2018a; Regrow Biosciences^®^, 2019). However, none of these therapies are approved so far by the FDA (FDA, 2019a) which demands compelling clinical evidence from reliable well-controlled trials, stronger policy compliance, and extensive premarket reviews (Marks et al., 2017; FDA, 2019b).

### Immune-Based Disorders

As discussed earlier (see section “Immunological Properties: A Paradigm”), MSCs possess immunomodulatory functions exhibited by their direct (cytokine-mediated) or indirect (T_*regs*_ modulation-mediated) inhibition of immune cells (Singer and Caplan, 2011; Haddad and Saldanha-Araujo, 2014). Those features are advantageous in treating immune-based disorders (Fitzsimmons et al., 2018). As such, therapies in this context exploit the immunomodulatory nature of MSC secretome which comprises NO, transforming growth factor-β, indoleamine 2,3-dioxygenase (IDO), prostaglandin E2, tumor necrosis factor-inducible gene 6 protein (TSG6), CCL-2, and PD-L1 among others. This immunomodulatory pool induces other immune cells to either modify/reprogram their response type (e.g., Th2 humoral-to-Th1 cellular immune response; dendritic cells (DCs) types 1 and 2 cytokine profile changes; and Th17-to-T_*reg*_ cell reprograming) (Aggarwal and Pittenger, 2005; Figueroa et al., 2012; Le Blanc and Mougiakakos, 2012) or generate immunosuppressive factors (Aggarwal and Pittenger, 2005; Han et al., 2012; Wei et al., 2013). As a result, MSCs are able to ameliorate pronounced immunity which is manifested in animal models of sepsis (Németh et al., 2009), autoimmune diseases (Constantin et al., 2009; Rafei et al., 2009), neurodegenerative disorders (Ma et al., 2013), and GvHD (Polchert et al., 2008). In particular, the earliest advancement in MSC immune-based clinical applications was recorded in GvHD, a serious complication arising from MHC-mismatched allografts affecting 20–70% of transplant recipients (Lee et al., 2003; Socié and Ritz, 2014). MSC administration in this setting drew the attention of the scientific community in 2004 after the remarkable response against resistant grade IV acute GvHD of the gut and liver in a 9-year-old boy who received the first transplantation of haploidentical MSCs (Le Blanc et al., 2004). Other phase II/III clinical trials followed, reporting variable levels of effectiveness (Introna et al., 2014; Van Der Wagen et al., 2014). In 2009, an industry-led large-scale phase III study evaluated the use of allogeneic BM-derived MSCs for treating steroid-refractory GvHD (NCT00366145) which occurs after failure of first-line corticosteroid treatment and affects 30–80% of graft recipients giving patients a 10–30% chance for long time survival (Luft et al., 2011). Despite the lack of a significant difference in clinical outcomes between placebo and treatment groups, a sub-group analysis led to the conditional approval of Prochymal^TM^ for the treatment of pediatric steroid-refractory GvHD (Kurtzberg et al., 2010; Martin et al., 2010) in Canada, New Zealand (2012) (Chisholm et al., 2019), and Japan (2015) (Sipp, 2015). Although the pass of Prochymal^TM^ was considered a breakthrough for MSC-based therapies, it remained largely unattainable in Canada and New Zealand due to strict prescription regulations and high manufacturing cost (USD 200,000) (Bersenev, 2016; Chisholm et al., 2019).

Moreover, an official approval for Darvadstrocel (Alofisel), an adipose human MSC injection, was granted by the European commission for the management of complex perianal fistulas in adult patients with mildly or non-active luminal Crohn’s disease (European Medicines Agency, 2018b; Panés et al., 2018). The approval emanated from a Phase III trial reporting that Darvadstrocel led to 50% combined remission, which was maintained after 1 year of treatment, in comparison with 34% in the control arm (Panés et al., 2016, 2018; Panes et al., 2017). Interestingly, several “orphan designation” approvals were granted by the European commission according to certain guidelines for the use of human MSCs in the treatment of GvHD, thromboangiitis obliterans (Buerger disease), and ALS (Yu et al., 2018; European Medicines Agency, 2019). Bader et al. (2018) the holders of one of the “orphan designations” in Germany (PEI.A.11748.01.1) for the treatment of steroid-resistant or treatment-refractory acute GvHD with their MSC preparation [MSC-Frankfurt am Main (MSC-FFM)], reported superior treatment outcomes in both adults and children as opposed to the limited efficacy of Prochymal^TM^ in children. According to their study, the effectiveness of MSC-FFM is due to donor selection in addition to strict collection and preparation processes (Bader et al., 2018), which yield adequate doses of MSCs with high batch-to-batch consistency (Elgaz et al., 2019). The distinguished data on MSC-FFM clearly elucidate the reasons behind the discrepancies (different survival rates and response levels to allogeneic MSC) and failures of other phase III clinical trials (Galipeau, 2013; Galipeau and Sensébé, 2018). In addition to the variation related to patient selection criteria (age, type, and disease clinical-grade), qualitative variabilities between MSC preparations play an important role. Lack of standardized manufacturing procedures such as donor heterogeneity, tissue origin variability (BM or adipose tissue), cell cryopreservation, culture expansion, administered dose and timing, heterogeneity of host inflammatory biomarkers, and immunogenicity are also among the variables (Galipeau, 2013; Squillaro et al., 2016; Galipeau and Sensébé, 2018). This also accords the fact that currently available MSC-based therapies for treating immune disorders – Remestemcel-L (Prochymal^®^) and TEMCELL^®^ for GvHD (JCR Pharmaceuticals Co, 2015; Locatelli et al., 2017), NeuroNata-R^®^ for ALS (Corestem, 2015), and Alofisel and Cupistem^®^ for Crohn’s anal fistula (ANTEROGEN, 2012; MilliporeSigma, 2017; European Medicines Agency, 2018a) – are still not FDA-approved despite their worldwide regulatory approval (Bernardo and Fibbe, 2013). Henceforth, further standardization of clinical-grade MSCs will better serve future clinical trials and facilitate international clinical approval. Equally important is expanding the knowledge of MSC polarization mechanisms and fates post-delivery (Duijvestein et al., 2010; Lechanteur et al., 2016; Russell et al., 2018; Grégoire et al., 2019).

## MSCs and Cancer

### Cancer Support or Suppression?

Cancer management using MSCs stems from the ability of these cells to home to tumors. Indeed, tumor tropism is a complex process involving multiple receptors and soluble factors. For example, SDF-1/C-X-C Motif Chemokine Receptor 4 (CXCR4), a chemokine/chemokine receptor axis involved in stem cell trafficking and cancer metastasis, plays a major role in MSC tumor infiltration (Phillips et al., 2003; Kucia et al., 2004). Tumor secretome induces MSC secretion of SDF-1, which activates in an autocrine fashion migratory signaling pathways (STAT3 and MAP kinase) and regulates cytoskeleton reorganization. According to certain studies, SDF-1 may also be part of tumor secretome (Gao et al., 2009; Lourenco et al., 2015). Overexpression of CXCR4 can, therefore, be considered therapeutically relevant due to its ability to augment MSC homing efficiency (Cheng et al., 2008). Macrophage Migration Inhibitory Factor (MIF), a pleotropic cytokine involved in multiple biological processes including tumor metastasis, is also implicated in MSC homing to tumors (Han et al., 2018). Like SDF-1, tumor-secreted MIF binds, among other receptors, to CXCR4 (G_*i*_-protein coupled receptor) and activates MAP kinase signaling pathway, eventually inducing MSC migration through upregulating cell motility genes. Other cytokines/chemokine ligands secreted by tumors also act as MSC attractants and may even trigger MSC expression of CXCR4 (Lourenco et al., 2015). In a similar fashion to CXCR4 overexpression, tumor homing can be amplified by engineering MSCs to overexpress specific tumor-binding receptors (Komarova et al., 2010). The homing process can be tracked with various *in vivo* optical- and fluorescent-based imaging techniques (Reagan and Kaplan, 2011). It is important to note that a recent clinical study showed that BM-derived MSCs failed to home to prostate cancer sites, an observation linked to the absence of inflammatory signals, which usually dictate MSC migration (Schweizer et al., 2019). These data might also question the innateness of unmodified allogeneic MSCs to home to tumors without reprograming (Serakinci and Cagsin, 2019). Therefore, additional clinical studies are necessary for validating the facts.

What’s more, current literature presents with data discrepancies as to whether unmodified MSCs support or suppress cancer growth. The first school reports that bearing the significant resemblance between mesenchymal tumor cells and MSCs in terms of proliferation/differentiation and pro-angiogenesis (Galiè et al., 2008), local mesenchymal progenitors or administered unmodified MSCs enhance cancer growth and metastasis, thus creating an “immunological sanctuary” in which tumor cells avoid immune surveillance (Hanahan and Weinberg, 2000; Krampera, 2011). These MSC properties of cancer support are originally licensed by tumor-infiltrating macrophages which establish a pro-inflammatory chemotactants-studded milieu (Coffelt et al., 2009; Rigo et al., 2010). This milieu evokes MSCs to (i) differentiate into highly proliferative myofibroblasts (Von Ahrens et al., 2017) and vascular cells (Peters et al., 2005), (ii) produce tumor-nurturing pro-angiogenic cytokines, miRNA, and exosomes (Roccaro et al., 2013; Zhang et al., 2013; Dong et al., 2018), (iii) secrete extracellular matrix-forming lysosomal oxidase (El-Haibi et al., 2012), (iv) provide a niche for malignant cells to thrive (Lin et al., 2019), and (v) adopt the immunomodulatory MSC2 phenotype (see section “Immunological Properties: A Paradigm”) (Patel et al., 2010). As previously mentioned, MSC2 further polarizes macrophages into the M2 phenotype which is pro-tumorigenic (Rivera-Cruz et al., 2017).

Contrastingly, the other school reports that MSCs are anti-tumorigenic. This observation is upheld by studies on various tumor types which demonstrate size/metastasis reduction or inhibition of proliferation upon MSC injection (Klopp et al., 2011). In this course, MSCs home to tumor sites and reinforce their anti-neoplastic effects by interacting with cancer cells *via* cell-cell adhesive proteins (e.g., *E*-cadherin, Khakoo et al., 2006) or releasing soluble factors (Maestroni et al., 1999) [e.g., dickkopf-1, a Wnt signaling inhibitor (Qiao et al., 2008; Zhu et al., 2009)] and anti-proliferative miRNA-containing vesicles (Reza et al., 2016). Molecularly, the effects are sustained by (i) interference with pro-survival/proliferation signaling pathways [e.g., protein kinase Akt (Khakoo et al., 2006; Dasari et al., 2010a) and Wnt/β-catenin (Secchiero et al., 2010)], (ii) activation of apoptotic pathways (e.g., Smac/DIABLO) (Dasari et al., 2010b; Reza et al., 2016), and (iii) cell cycle arrest in G0/G1 phase (Lu et al., 2008; Cousin et al., 2009). The net signaling transduced favors an upregulation of cell cycle modulators (e.g., p21) and pro-apoptotic proteins (e.g., caspase 3, caspase 9, BAX) (Lu et al., 2008; Reza et al., 2016), opposed by a downregulation of anti-apoptotic mediators (e.g., XIAP, BCL2) (Dasari et al., 2010a, b; Reza et al., 2016). Besides, MSCs can inhibit neo-angiogenesis by forming gap junctions with endothelial cells and supplying them with reactive oxygen species, which induce their apoptosis (Otsu et al., 2009; Secchiero et al., 2010).

The inconsistencies between both schools are attributed to multiple factors including MSC source/preparation, administration timing/dose, polarization, and tumor variability (Klopp et al., 2011).

### Therapeutic Management

Mesenchymal stem cell properties of tumor tropism and non-immunogenicity were used in antitumor research. The methodology involved transforming MSCs into a therapeutic platform able to inherently engraft in tumor architecture and genetically produce recombinant antitumor or antitumor immunity-driving molecules. Examples include tumor necrosis factor-related apoptosis-inducing ligand (TRAIL) (Loebinger et al., 2009), C-X3-C motif chemokine ligand 1 (CX3CL1) (Xin et al., 2009), IFN-β (Studeny et al., 2002; Ren et al., 2008b), IFN-α (Ren et al., 2008a), IFN-γ (Li X. et al., 2006), IL-2 (Nakamura et al., 2004), and (modified) IL-12 (Chen X. et al., 2008; Seo et al., 2011). For example, a study by Li X. et al. (2006) showed that autologous MSCs derived from a leukemic patient then engineered to generate IFN-γ significantly inhibit the proliferation of leukemia cell lines and induce their apoptosis. In the same context, other genetic engineering-based methods include MSCs which express (i) replicative adenoviruses that infect cancer cells and induce oncolysis (e.g., ICOVIR5, Ad5-DNX-2401), (i) therapeutic gene-incorporating retroviral vectors, and (iii) suicidal gene-incorporating vectors. However, these efficient interventions confer toxicity and require simultaneous anti-retroviral drugs administration (Uchibori et al., 2009; Loebinger and Janes, 2010). Researchers also fostered MSC-based vehicles independent of genetic engineering. Those exploit the innateness of MSCs to uptake drugs *in vitro* allegedly through Golgi-derived vesicles (drug uptake mechanisms are insufficiently characterized and are not confined to MSCs, Girdlestone, 2016). Although their drug sensitivity varies according to cell source, MSCs rapidly internalize sufficient drug molecules, such that following MSC administration to animal models, captured drugs are slowly and sufficiently released in their original form (active or prodrug) into tumor vicinity (Pessina et al., 2011; Bonomi et al., 2013; Coccè et al., 2017). Likewise, MSCs can be loaded with prodrugs to effectively inhibit cancer growth (Levy et al., 2016). These observations led to few human cancer management studies, which are still taking their baby steps toward clinical efficacy. For example, in a phase I/II study (TREAT-ME1), autologous MSCs were isolated from patients according to GMP standards and transfected with replication-incompetent retroviral vectors to generate MSC_apceth_101, an investigational medicinal product containing a therapeutic promoter-gene construct aimed to treat advanced gastrointestinal tumors. The trial, however, did not advance to therapeutic confirmatory phase III due to adverse events and lack of disease amelioration (EudraCT Number 2012-003741-15) (Niess et al., 2015). Other challenges in MSC-based anticancer treatment are, paradoxically, cancer enhancement (Karnoub et al., 2007) even with induced anti-tumor immunity (vaccination) (Krampera et al., 2007) as well as insufficient cell homing to tumors to guarantee efficient delivery of therapeutic agents (Schweizer et al., 2019).

### Cancer Vaccination

Vaccination is a robust, safe, and cost-effective preventative or therapeutic method against pathogenic diseases (Tomchuck et al., 2012). While therapeutic vaccines induce cell-mediated immunity and are used to eliminate existing pathogens/lesions or prevent their progression, preventative vaccines trigger humoral immunity (serum antibody generation) for prophylaxis of futuristic pathogens/lesions (Nayereh and Khadem, 2012).

Traditionally, vaccine development employs the attenuation or inactivation of a pathogen to create long-term immune memory and/or mount a durable immune response against intact pathogens. Although efficient against several mortal diseases (smallpox, diphtheria, polio, measles), vaccines still lack in offering protection against their ilk (HIV, malaria, common cold, tuberculosis) due to robust microbial antigen shifting or difficult intracellular pathogen accessibility which complicates the selection of target antigens. In addition to intact antigenic peptides, alternative vaccines exist, such as *in situ* antigen production or presentation using plasmid vectors (DNA) and antigen-pulsed host cells (APCs, MSCs). However, they have not yet achieved any clinical benefits, mainly due to their low immunogenicity (MacGregor et al., 1998; Tomchuck et al., 2012; Hobernik and Bros, 2018).

The notion of cancer vaccination, an increasingly active research topic, stems from the inherent role of the immune system to eliminate cancer cells and the possibility thereof to develop immune enhancing therapies to adequately eradicate tumors (Butterfield, 2015). For this purpose, synthetic neo-antigens (Ott et al., 2017) as well as DNA- and cellular-based platforms exercising foreign antigen/cytokine production or expression have been used to devise tumor epitope-specific vaccines or instigate anti-tumor T-cell reactivity *in vitro*. This strategy was efficient as an *in vivo* cancer immunotherapy, especially if the regimen involves immune-checkpoint blocking antibodies to enhance effector T cells function by blocking their inhibitory receptors (PD-1 and CTLA-4) (Schumacher and Schreiber, 2015; Wraith, 2017).

Among the best candidates for cellular-based vaccine platforms, DCs are especially efficient APCs and primers of immune responses (Guéry and Adorini, 1995; Janikashvili et al., 2010; Le et al., 2010; Palucka and Banchereau, 2013). Plus, DCs are considered natural adjuvants as they can modulate and interconnect innate adaptive immune responses through their surface molecules and secretome (Mellman and Steinman, 2001; Steinman, 2001). In clinic, Sipuleucel-T, branded as Provenge, is the first and only FDA-approved DC vaccine for the treatment of asymptomatic or minimally symptomatic metastatic and castration-resistant prostate cancer (Small et al., 2006; Higano et al., 2010; Anassi and Ndefo, 2011; Cheever and Higano, 2011). However, other attempts at DC vaccine introduction in animal and clinical studies faced more complications than anticipated, demonstrating immense variation in reported outcomes (Le et al., 2010; Robson et al., 2010; Mantia-Smaldone and Chu, 2013). Reasons for such clinical discrepancies can be attributed to DC non-standardized *ex vivo* preparation and administration protocols which entail multiple variabilities at the level of (i) DCs source/phenotype, (ii) DCs maturation stimulus used, (iii) nature/procedure for antigen loading, (iv) route of administration, and (v) dose (Nicolette et al., 2007). Besides, their high production cost, low production grade, limited effectiveness, and immunogenicity hamper their clinical acceptance and advancement (Chambers and Neumann, 2011; Bhargava et al., 2012; Datta et al., 2014; Jarosławski and Toumi, 2015; Wei et al., 2015). Therefore, the search for other cellular-based vaccines with potentially better performance in these criteria was necessary, and so MSCs came forth as a fit vaccine platform in this regard.

**FIGURE 1:**
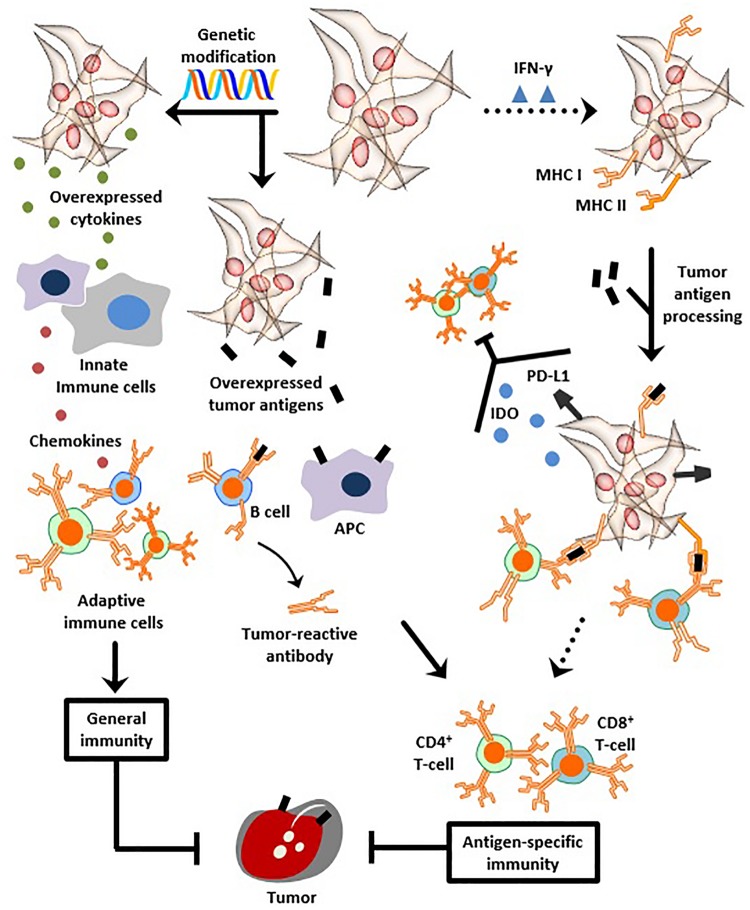
MSCs as anti-cancer vaccines. MSCs can be genetically modified to overexpress cytokines to instigate innate and adaptive immunity, as a means to protect against neoplasms. Genetic modification can be also used to overexpress tumor antigens and instill anti-tumor humoral and cellular immunity. Likewise, dose- and time-dependent exposure to IFN-γ transforms MSCs, albeit transiently, into APCs capable of providing antigen-specific immune protection. This occurs through induction of MHC class I and II expression, followed by tumor antigen processing and MHC-mediated presentation to T-cells. Despite IFN-γ-induced antigen presentation, other observations report that MSCs simultaneously up-regulate PD-L1 and secrete IDO, both of which inhibit T-cells. Henceforth, overcoming the transient and temporary antigen presenting properties of IFN-γ-exposed MSCs is necessary to achieve vigorous stability and abundance of presented neoantigens, thus helping to create a clinically efficient anti-cancer vaccine.

MSCs can elicit general and/or antigen-specific immunity, without being immunogenic themselves, depending on three assets (Figure 1). First, MSCs are context-specific pro-inflammatory (see Section “Immunological Properties: A Paradigm”), a property which ultimately renders them enhancers of humoral and cellular immunity. Second, MSCs are genetically modifiable, thereby representing suitable vehicles for producing and secreting cytokines or soluble antigens which evoke robust immune responses. A report by Wei et al. (2011) follows this scenario albeit to a certain extent. In the details, the group devised a combined vaccine consisting of a fusion protein vaccine which targets E7 tumor antigen and immortalized human MSCs designed to express E7 antigens. Compared to the fusion protein vaccine alone, the combined vaccine elicited significantly stronger tumoricidal immunological reactions when administered to subcutaneous and lung metastasis mice models. The authors propose that those effects ensue after the tagging of tumor cells with E7 antigens released by infiltrating MSCs along with the instigation of humoral immunity by the fusion protein vaccine. The generated anti-E7 antibodies were, therefore, able to recognize tumors and eventually suppress their growth (Wei et al., 2011). Third and most importantly, MSCs can act as APCs capable of processing and presenting exogenous antigens to activate immune cells; this asset surfaces in response to IFN-γ treatment which induces MSC expression of MHCI/II molecules (Majumdar et al., 2003; Stagg et al., 2006; François et al., 2009; Tomchuck et al., 2012; van Megen et al., 2019). This property was exploited in cancer vaccination studies, which are hitherto limited. For instance, mice vaccinated with IFN-γ-licensed MSCs pulsed with ovalbumin antigen are completely protected when challenged with ovalbumin-expressing E.G7 lymphatic tumors (Stagg et al., 2006; François et al., 2009; Stagg and Galipeau, 2013). Protection against tumors using IFN-γ-treated MSCs is conferred through MHC I upregulation, MHC II induction, and, in part, through the upregulation of the antigen processing machinery responsible for translocation of processed antigens into the ER before trafficking toward the plasma membrane. Overall, this enhances antigen presentation to CD4^+^ T-cells (MHC II-restricted) and cross-presentation to CD8^+^ T-cells (MHC I-restricted), both of which respond by increased activation and proliferation (François et al., 2009). Another study further shows that the strong anti-tumorigenic immune responses evoked by IFN-γ-treated MSCs involve CD80 (co-stimulatory molecule) and MHC class II- but not class I-mediated antigen presentation, albeit the induction of strong CD8^+^ T-cell responses *in vivo*. The authors argue that antigen cross-presentation which is not observed *in vitro* can develop *in vivo* not in MSCs themselves but in other host APCs which can acquire their antigens from MSCs in a process termed cross-priming (Stagg et al., 2006). Paralleling, a recent study reports that although IFN-γ-licensed human MSCs uptake and process antigens and upregulate MHC class II but not CD80, their pro-inflammatory secretome remains intact. Importantly, the study also shows that despite their IFN-γ-induced antigen presentation, MSCs inhibit autoreactive T-cells, an observation associated with PD-L1 upregulation and IDO secretion (Figure 1). The inhibitory effect even lasted beyond the removal of MSCs and the introduction of activation signals (antigen-pulsed DCs) (van Megen et al., 2019). However, in another report, IFN-γ-induced upregulation of PD-L1 on antigen-presenting MSCs is believed to be tied to T-cell induction rather than inhibition (Stagg et al., 2006). This discrepancy adds to the many layers of MSC character.

A side note, more prevalent is the therapeutic induction of general rather than antigen-specific anti-tumor immunity (Wei et al., 2011). This is evident in the variety of researched MSC vaccines which, as mentioned in Section “Cancer Support or Suppression?”, genetically express recombinant immunostimulatory molecules (Studeny et al., 2002; Nakamura et al., 2004; Li X. et al., 2006; Chen X. et al., 2008; Ren et al., 2008a, b; Xin et al., 2009; Seo et al., 2011). Furthermore, while prophylactic MSC-based anti-cancer vaccines are more strenuous to devise compared to their therapeutic counterparts (tumor antigens have unique expression patterns), prophylactic MSC-based anti-microbial vaccines attain their purpose of triggering antigen-specific humoral and adaptive immunity against, respectively, HIV and tetanus (Tomchuck et al., 2012). In sharp contrast, the clinical knowledge available thus far on MSCs as cancer vaccines is, unfortunately, insufficient to advance further their proof of concept. Table 2 demonstrates the only registered human studies utilizing MSC-based anti-cancer therapeutic vaccines.

**TABLE 2:** The clinical trials assessing MSC-based vaccines for cancer treatment.

**NCT**	**Study phase**	**Start date**	**Vaccine properties**	**Cancer type**	**Results/Status**
02079324	1	2014	aka GX-051, IL-12-expressing, induces IFN-γ production and subsequently cellular immunity	Head and neck	Unknown
02530047	1	2016	IFN-β-expressing, immunostimulatory	Ovarian	Completed, no disclosed results

## Conclusion and Future Recommendations

In summary, due to their regenerative abilities, immunomodulation, tumor homing, and multiple other advantages, MSCs have demonstrated unprecedented potential in cellular therapy *in vivo*, specifically against immunological, degenerative, and cancer pathologies. Therefrom, their international clinical approval is a matter of time. Likewise, the growing notion of MSC vaccination has demonstrated promising potential for cancer prophylaxis or therapy, despite the scarcity of relevant clinical data. Reflecting on the reasons behind this, it is legit to say that MSC vaccine-based cancer research requires further understanding not only of the intervention itself but also of the multiple intricacies characterizing the interplay between MSCs and both tumors and immune cells. More specifically, an efficient MSC-based anti-cancer vaccine first needs to overcome the transient and temporary antigen presenting properties observed after IFN-γ exposure. As mentioned earlier, our current understanding of MSCs as APCs is indispensable of the dose-dependent temporary exposure to IFN-γ alongside the phenotypic responses arising therefrom (Figure 1) (Chan et al., 2006).

Other immunomodulatory observations upon IFN-γ licensing of antigen-pulsed MSCs are also recorded (van Megen et al., 2019). For example, IFN-γ treatment is associated with the upregulation of B7-H1 (PD-L1) (Krampera et al., 2006). These intricacies show that we need to understand the antigen presenting properties of MSCs beyond IFN-γ. Bypassing this conditional APC state thus warrants vigorous stability and abundance of MHCI/II-presented neo-antigens. Also, sufficient molecular knowledge of protein translation, proteasome degradation of proteins, endoplasmic reticulum transport, and affinity for MHC molecules – all in direct link to antigen presentation – is necessary (Schumacher and Schreiber, 2015). Equally important is realizing cancer complexity and the burden of tumor stromal cells in oncological settings (Brahmer et al., 2012; Joyce and Fearon, 2015). Since tumor stromal cells induce massive alterations in local metabolome and secretome profiles and are thought to ensnare CD8^+^ T cells and other APCs (Joyce and Fearon, 2015; Hammerich et al., 2019) in the tumor microenvironment, their contribution to immune suppression, evasion, and unresponsiveness to immune-checkpoint blockers (Brahmer et al., 2012) should be investigated more in depth. Consequently, surpassing these obstacles, perhaps by instilling potent and stable antigen cross-presentation properties in properly treated MSCs, as well as ensuring that adaptive immunity is actively triggered and always one step ahead of tumor intelligence, will allow us to harness the full capacity of MSCs as robust APCs.

## Author Contributions

RS, AE-K, JA, and MR contributed in writing the review.

## Conflict of Interest

RS is the founder of IntelliStem Technologies Inc. (Toronto, ON, Canada). The remaining authors declare that the research was conducted in the absence of any commercial or financial relationships that could be construed as a potential conflict of interest.

## Abbreviations

ALS: amyotrophic lateral sclerosis
APC(s): antigen secreting cell(s)
BM: bone marrow
CX3CL1: C-X 3-C motif chemokine ligand 1
CXCR4: C-X -C Motif Chemokine Receptor 4
DC(s): dendritic cell(s)
GMP: good manufacturing practice
GvHD: graft-versus-host disease
IDO: indoleamine 2,3-dioxygenase
IFN: interferon
IL: interleukin
ISCT: International Society for Cellular Therapy
MAP: mitogen activated protein
MI: myocardial infarction
MIF: Migratory Inhibitory Factor
miRNA: microRNA
MSC(s): mesenchymal stem cell(s)
NK: natural killer
NO: nitric oxide
PD-L1: programed death-ligand 1
SDF-1: stromal-derived factor-1
TLRs: toll-like receptors
TRAIL: tumor necrosis factor-related apoptosis-inducing ligand
T_*reg*_: regulatory T cell
TSG6: tumor necrosis factor-inducible gene 6 protein.

## References

[B1] Aggarwal S. Pittenger M. F. (2005). Human mesenchymal stem cells modulate allogeneic immune cell responses. *Blood* 105 1815–1822. 10.1182/blood-2004-04-1559 15494428

[B2] Allers C. Sierralta W. D. Neubauer S. Rivera F. Minguell J. J. Conget P. A. (2004). Dynamic of distribution of human bone marrow-derived mesenchymal stem cells after transplantation into adult unconditioned mice. *Transplantation* 78 503–508. 10.1097/01.TP.0000128334.93343.B3 15446307

[B3] Al-Nbaheen M. vishnubalaji R. Ali D. Bouslimi A. Al-Jassir F. Megges M. (2013). Human stromal (mesenchymal) stem cells from bone marrow, adipose tissue and skin exhibit differences in molecular phenotype and differentiation potential. *Stem Cell Rev. Rep.* 9 32–43. 10.1007/s12015-012-9365-8 22529014 PMC3563956

[B4] Amado L. C. Saliaris A. P. Schuleri K. H. St John M. Xie J.-S. Cattaneo S. (2005). Cardiac repair with intramyocardial injection of allogeneic mesenchymal stem cells after myocardial infarction. *Proc. Natl. Acad. Sci. U.S.A.* 102 11474–11479. 10.1073/pnas.0504388102 16061805 PMC1183573

[B5] Anassi E. Ndefo U. A. (2011). Sipuleucel-T (provenge) injection: the first immunotherapy agent (vaccine) for hormone-refractory prostate cancer. *Pharm. Ther.* 36 197–202. PMC3086121 21572775

[B6] Anger F. Camara M. Ellinger E. Germer C.-T. Schlegel N. Otto C. (2019). Human mesenchymal stromal cell-derived extracellular vesicles improve liver regeneration after ischemia reperfusion injury in mice. *Stem Cells Dev.* 28 1451–1462. 10.1089/scd.2019.0085 31495270

[B7] Ansari S. Chen C. Hasani-Sadrabadi M. M. Yu B. Zadeh H. H. Wu B. M. (2017). Hydrogel elasticity and microarchitecture regulate dental-derived mesenchymal stem cell-host immune system cross-talk. *Acta Biomater.* 60 181–189. 10.1016/j.actbio.2017.07.017 28711686 PMC5581234

[B8] ANTEROGEN (2012). *Cupistem^®^ Injection.* Available at: http://anterogen.com/main/en/sub02_01.html?type=1 (accessed September 15, 2019).

[B9] Asahara T. Takahashi T. Masuda H. Kalka C. Chen D. Iwaguro H. (1999). VEGF contributes to postnatal neovascularization by mobilizing bone marrow-derived endothelial progenitor cells. *EMBO J.* 18 3964–3972. 10.1093/emboj/18.14.3964 10406801 PMC1171472

[B10] Bader P. Kuçi Z. Bakhtiar S. Basu O. Bug G. Dennis M. (2018). Effective treatment of steroid and therapy-refractory acute graft-versus-host disease with a novel mesenchymal stromal cell product (MSC-FFM). *Bone Marrow Transplant.* 53 852–862. 10.1038/s41409-018-0102-z 29379171 PMC6039391

[B11] Bernardo M. E. Fibbe W. E. (2013). Mesenchymal stromal cells: sensors and switchers of inflammation. *Cell Stem Cell* 13 392–402. 10.1016/J.STEM.2013.09.006 24094322

[B12] Bernardo M. E. Zaffaroni N. Novara F. Cometa A. M. Avanzini M. A. Moretta A. (2007). Human bone marrow–derived Mesenchymal stem cells do not undergo transformation after long-term *In vitro* culture and do not exhibit telomere maintenance mechanisms. *Cancer Res.* 67 9142–9149. 10.1158/0008-5472.CAN-06-4690 17909019

[B13] Bersenev A. (2016). *Why Price for Cell/Gene Therapy Products is So High?* Available at: https://celltrials.info/2016/09/06/pricing/ (accessed October 21, 2019).

[B14] Bhargava A. Mishra D. Banerjee S. Mishra P. K. (2012). Dendritic cell engineering for tumor immunotherapy: from biology to clinical translation. *Immunotherapy* 4 703–718. 10.2217/imt.12.40 22853757

[B15] Bianco P. Robey P. G. Simmons P. J. (2008). Mesenchymal stem cells: revisiting history, concepts, and assays. *Cell Stem Cell* 2 313–319. 10.1016/j.stem.2008.03.002 18397751 PMC2613570

[B16] BioInformant (2019). *Mesenchymal Stem Cells - Advances & Applications.* Available at: https://bioinformant.com/product/mesenchymal-stem-cells-advances-and-applications/ (accessed September 15, 2019).

[B17] Bonomi A. Coccè V. Cavicchini L. Sisto F. Dossena M. Balzarini P. (2013). Adipose tissue-derived stromal cells primed in vitro with paclitaxel acquire anti-tumor activity. *Int. J. Immunopathol. Pharmacol.* 26 33–41. 10.1177/03946320130260S105 24046947

[B18] Brahmer J. R. Tykodi S. S. Chow L. Q. M. Hwu W.-J. Topalian S. L. Hwu P. (2012). Safety and activity of Anti–PD-L1 antibody in patients with advanced cancer. *N. Engl. J. Med.* 366 2455–2465. 10.1056/NEJMoa1200694 22658128 PMC3563263

[B19] Butterfield L. H. (2015). Cancer vaccines. *BMJ* 350:h988. 10.1136/bmj.h988 25904595 PMC4707521

[B20] Cai B. Tan X. Zhang Y. Li X. Wang X. Zhu J. (2015). Mesenchymal stem cells and cardiomyocytes interplay to prevent myocardial hypertrophy. *Stem Cells Transl. Med.* 4 1425–1435. 10.5966/sctm.2015-0032 26586774 PMC4675503

[B21] Cai M. Shen R. Song L. Lu M. Wang J. Zhao S. (2016). Bone marrow mesenchymal stem cells (BM-MSCs) improve heart function in swine myocardial infarction model through paracrine effects. *Sci. Rep.* 6:28250. 10.1038/srep28250 27321050 PMC4913323

[B22] Campeau P. M. Rafei M. Boivin M.-N. Sun Y. Grabowski G. A. Galipeau J. (2009). Characterization of Gaucher disease bone marrow mesenchymal stromal cells reveals an altered inflammatory secretome. *Blood* 114 3181–3190. 10.1182/blood-2009-02-205708 19587377 PMC2925728

[B23] Caplan A. I. (1991). Mesenchymal stem cells. *J. Orthop. Res.* 9 641–650. 10.1002/jor.1100090504 1870029

[B24] Caplan A. I. Dennis J. E. (2006). Mesenchymal stem cells as trophic mediators. *J. Cell. Biochem.* 98 1076–1084. 10.1002/jcb.20886 16619257

[B25] Chambers J. D. Neumann P. J. (2011). Listening to provenge - What a costly cancer treatment says about future medicare policy. *N. Engl. J. Med.* 364 1687–1689. 10.1056/NEJMp1103057 21470004

[B26] Chan J. L. Tang K. C. Patel A. P. Bonilla L. M. Pierobon N. Ponzio N. M. (2006). Antigen-presenting property of mesenchymal stem cells occurs during a narrow window at low levels of interferon-gamma. *Blood* 107 4817–4824. 10.1182/blood-2006-01-0057 16493000 PMC1895812

[B27] Cheever M. A. Higano C. S. (2011). PROVENGE (sipuleucel-T) in prostate cancer: the first FDA-approved therapeutic cancer vaccine. *Clin. Cancer Res.* 17 3520–3526. 10.1158/1078-0432.CCR-10-3126 21471425

[B28] Chen L. Tredget E. E. Wu P. Y. G. Wu Y. (2008). Paracrine factors of Mesenchymal stem cells recruit macrophages and endothelial lineage cells and enhance wound healing. *PLoS One* 3:e1886. 10.1371/journal.pone.0001886 18382669 PMC2270908

[B29] Chen Q. Shou P. Zheng C. Jiang M. Cao G. Yang Q. (2016). Fate decision of mesenchymal stem cells: adipocytes or osteoblasts? *Cell Death Differ.* 23 1128–1139. 10.1038/cdd.2015.168 26868907 PMC4946886

[B30] Chen X. Lin X. Zhao J. Shi W. Zhang H. Wang Y. (2008). A tumor-selective biotherapy with prolonged impact on established metastases based on cytokine gene-engineered MSCs. *Mol. Ther.* 16 749–756. 10.1038/MT.2008.3 18362930

[B31] Cheng Z. Ou L. Zhou X. Li F. Jia X. Zhang Y. (2008). Targeted migration of Mesenchymal stem cells modified with CXCR4 Gene to infarcted myocardium improves cardiac performance. *Mol. Ther.* 16 571–579. 10.1038/sj.mt.6300374 18253156

[B32] Chisholm J. Ruff C. Viswanathan S. (2019). Current state of health Canada regulation for cellular and gene therapy products: potential cures on the horizon. *Cytotherapy* 21 686–698. 10.1016/j.jcyt.2019.03.005 31196821

[B33] Choi J.-J. Yoo S.-A. Park S.-J. Kang Y.-J. Kim W.-U. Oh I.-H. (2008). Mesenchymal stem cells overexpressing interleukin-10 attenuate collagen-induced arthritis in mice. *Clin. Exp. Immunol.* 153 269–276. 10.1111/j.1365-2249.2008.03683.x 18713142 PMC2492894

[B34] Chung E. Son Y. (2014). Crosstalk between mesenchymal stem cells and macrophages in tissue repair. *Tissue Eng. Regen. Med.* 11 431–438. 10.1007/s13770-014-0072-1

[B35] Coccè V. Farronato D. Brini A. T. Masia C. Giannì A. B. Piovani G. (2017). Drug loaded gingival Mesenchymal stromal cells (GinPa-MSCs) inhibit in vitro proliferation of oral squamous cell carcinoma. *Sci. Rep.* 7:9376. 10.1038/s41598-017-09175-4 28839168 PMC5571047

[B36] Coffelt S. B. Marini F. C. Watson K. Zwezdaryk K. J. Dembinski J. L. LaMarca H. L. (2009). The pro-inflammatory peptide LL-37 promotes ovarian tumor progression through recruitment of multipotent mesenchymal stromal cells. *Proc. Natl. Acad. Sci. U.S.A.* 106 3806–3811. 10.1073/pnas.0900244106 19234121 PMC2656161

[B37] Conforti A. Starc N. Biagini S. Tomao L. Pitisci A. Algeri M. (2016). Resistance to neoplastic transformation of ex-vivo expanded human mesenchymal stromal cells after exposure to supramaximal physical and chemical stress. *Oncotarget* 7 77416–77429. 10.18632/oncotarget.12678 27764806 PMC5363595

[B38] Constantin G. Marconi S. Rossi B. Angiari S. Calderan L. Anghileri E. (2009). Adipose-derived Mesenchymal stem cells ameliorate chronic experimental autoimmune encephalomyelitis. *Stem Cells* 27 2624–2635. 10.1002/stem.194 19676124

[B39] Corestem (2015). *ALS (NeuroNata-R^®^).* Available at: http://corestem.com/en/m21.php (accessed September 15, 2019).

[B40] Cousin B. Ravet E. Poglio S. De Toni F. Bertuzzi M. Lulka H. (2009). Adult stromal cells derived from human adipose tissue provoke pancreatic cancer cell death both in vitro and in vivo. *PLoS One* 4:e6278. 10.1371/journal.pone.0006278 19609435 PMC2707007

[B41] Crisostomo P. R. Wang Y. Markel T. A. Wang M. Lahm T. Meldrum D. R. (2008). Human mesenchymal stem cells stimulated by TNF-α, LPS, or hypoxia produce growth factors by an NFκB- but not JNK-dependent mechanism. *Am. J. Physiol. Physiol.* 294 C675–C682. 10.1152/ajpcell.00437.2007 18234850

[B42] Dasari V. R. Kaur K. Velpula K. K. Gujrati M. Fassett D. Klopfenstein J. D. (2010b). Upregulation of PTEN in glioma cells by cord blood mesenchymal stem cells inhibits migration via downregulation of the PI3K/Akt pathway. *PLoS One* 5:e10350. 10.1371/journal.pone.0010350 20436671 PMC2859936

[B43] Dasari V. R. Velpula K. K. Kaur K. Fassett D. Klopfenstein J. D. Dinh D. H. (2010a). Cord blood stem cell-mediated induction of apoptosis in glioma downregulates X-linked inhibitor of apoptosis protein (XIAP). *PLoS One* 5:e11813. 10.1371/journal.pone.0011813 20676365 PMC2911373

[B44] Datta J. Terhune J. H. Lowenfeld L. Cintolo J. A. Xu S. Roses R. E. (2014). Optimizing dendritic cell-based approaches for cancer immunotherapy. *Yale J. Biol. Med.* 87 491–518. 25506283 PMC4257036

[B45] De Bari C. Dell’Accio F. Tylzanowski P. Luyten F. P. (2001). Multipotent mesenchymal stem cells from adult human synovial membrane. *Arthrit. Rheum.* 44 1928–1942. 10.1002/1529-0131(200108) 11508446

[B46] De Becker A. Van Riet I. (2016). Homing and migration of mesenchymal stromal cells: how to improve the efficacy of cell therapy? *World J. Stem Cells* 8 73–87. 10.4252/wjsc.v8.i3.73 27022438 PMC4807311

[B47] De Ugarte D. A. Morizono K. Elbarbary A. Alfonso Z. Zuk P. A. Zhu M. (2003). Comparison of multi-lineage cells from human adipose tissue and bone marrow. *Cells Tissues Organs* 174 101–109. 10.1159/000071150 12835573

[B48] Devine S. M. Bartholomew A. M. Mahmud N. Nelson M. Patil S. Hardy W. (2001). Mesenchymal stem cells are capable of homing to the bone marrow of non-human primates following systemic infusion. *Exp. Hematol.* 29 244–255. 10.1016/S0301-472X(00)00635-4 11166464

[B49] Ding D.-C. Shyu W.-C. Lin S.-Z. (2011). Mesenchymal stem cells. *Cell Transplant.* 20 5–14. 10.3727/096368910X 21396235

[B50] Dominici M. Le Blanc K. Mueller I. Slaper-Cortenbach I. Marini F. Krause D. S. (2006). Minimal criteria for defining multipotent mesenchymal stromal cells. The international society for cellular therapy position statement. *Cytotherapy* 8 315–317. 10.1080/14653240600855905 16923606

[B51] Dong L. Pu Y. Zhang L. Qi Q. Xu L. Li W. (2018). Human umbilical cord mesenchymal stem cell-derived extracellular vesicles promote lung adenocarcinoma growth by transferring miR-410. *Cell Death Dis.* 9:218. 10.1038/s41419-018-0323-5 29440630 PMC5833395

[B52] Duffy M. M. Ritter T. Ceredig R. Griffin M. D. (2011). Mesenchymal stem cell effects on T-cell effector pathways. *Stem Cell Res. Ther.* 2:34. 10.1186/scrt75 21861858 PMC3219065

[B53] Duijvestein M. Vos A. C. W. Roelofs H. Wildenberg M. E. Wendrich B. B. Verspaget H. W. (2010). Autologous bone marrow-derived mesenchymal stromal cell treatment for refractory luminal Crohn’s disease: results of a phase I study. *Gut* 59 1662–1669. 10.1136/gut.2010.215152 20921206

[B54] Eggenhofer E. Hoogduijn M. J. (2012). Mesenchymal stem cell-educated macrophages. *Transplant. Res.* 1:12. 10.1186/2047-1440-1-12 23369493 PMC3560988

[B55] Elgaz S. Kuçi Z. Kuçi S. Bönig H. Bader P. (2019). Clinical use of mesenchymal stromal cells in the treatment of acute graft-versus-host disease. *Transfus. Med. Hemother.* 46 27–34. 10.1159/000496809 31244579 PMC6558336

[B56] El-Haibi C. P. Bell G. W. Zhang J. Collmann A. Y. Wood D. Scherber C. M. (2012). Critical role for lysyl oxidase in mesenchymal stem cell-driven breast cancer malignancy. *Proc. Natl. Acad. Sci. U.S.A.* 109 17460–17465. 10.1073/pnas.1206653109 23033492 PMC3491529

[B57] Eliopoulos N. Galipeau J. (2002). Green fluorescent protein in retroviral vector constructs as marker and reporter of gene expression for cell and gene therapy applications. *Methods Mol. Biol.* 183 353–371. 10.1385/1-59259-280-5:353 12136771

[B58] Erokhin V. V. Vasil’eva I. A. Konopliannikov A. G. Chukanov V. I. Tsyb A. F. Bagdasarian T. R. (2008). Systemic transplantation of autologous mesenchymal stem cells of the bone marrow in the treatment of patients with multidrug-resistant pulmonary tuberculosis. *Probl. Tuberk. Bolezn. Legk.* 10 3–6. 19086127

[B59] European Medicines Agency (2017). *Spherox.* Available at: https://www.ema.europa.eu/en/medicines/human/EPAR/spherox (accessed September 15, 2019).

[B60] European Medicines Agency (2018a). *Alofisel.* Available at: https://www.ema.europa.eu/en/medicines/human/EPAR/alofisel (accessed September 15, 2019).

[B61] European Medicines Agency (2018b). *EPAR Summary for the Public.* Amsterdam: European Medicines Agency.

[B62] European Medicines Agency (2019). Available at: https://www.ema.europa.eu/en (accessed October 10, 2019).

[B63] Ezquer F. E. Ezquer M. E. Vicencio J. M. Calligaris S. D. (2017). Two complementary strategies to improve cell engraftment in mesenchymal stem cell-based therapy: Increasing transplanted cell resistance and increasing tissue receptivity. *Cell Adh. Migr.* 11:110. 10.1080/19336918.2016.1197480 27294313 PMC5308221

[B64] FDA (2019a). *Approved Cellular and Gene Therapy Products.* Available at: https://www.fda.gov/vaccines-blood-biologics/cellular-gene-therapy-products/approved-cellular-and-gene-therapy-products (accessed September 15, 2019).

[B65] FDA (2019b). *Statement from FDA Commissioner Scott Gottlieb, M.D. on the FDA’s New Policy Steps and Enforcement Efforts to Ensure Proper Oversight Of Stem Cell Therapies and Regenerative Medicine.* Available at: https://www.fda.gov/news-events/press-announcements/statement-fda-commissioner-scott-gottlieb-md-fdas-new-policy-steps-and-enforcement-efforts-ensure (accessed September 15, 2019).

[B66] Figueroa F. E. Carrión F. Villanueva S. Khoury M. (2012). Mesenchymal stem cell treatment for autoimmune diseases: a critical review. *Biol. Res.* 45 269–277. 10.4067/S0716-97602012000300008 23283436

[B67] Fitzsimmons R. E. B. Mazurek M. S. Soos A. Simmons C. A. (2018). Mesenchymal stromal/stem cells in regenerative medicine and tissue engineering. *Stem Cells Int.* 2018 8031718. 10.1155/2018/8031718 30210552 PMC6120267

[B68] Fontaine C. Cousin W. Plaisant M. Dani C. Peraldi P. (2008). Hedgehog signaling alters adipocyte maturation of human mesenchymal stem cells. *Stem Cells* 26 1037–1046. 10.1634/stemcells.2007-0974 18258719

[B69] Fouillard L. Bensidhoum M. Bories D. Bonte H. Lopez M. Moseley A.-M. (2003). Engraftment of allogeneic mesenchymal stem cells in the bone marrow of a patient with severe idiopathic aplastic anemia improves stroma. *Leukemia* 17 474–476. 10.1038/sj.leu.2402786 12592355

[B70] François M. Romieu-Mourez R. Stock-Martineau S. Boivin M.-N. N. Bramson J. L. Galipeau J. (2009). Mesenchymal stromal cells cross-present soluble exogenous antigens as part of their antigen-presenting cell properties. *Blood* 114 2632–2638. 10.1182/blood-2009-02-207795 19654411

[B71] François S. Bensidhoum M. Mouiseddine M. Mazurier C. Allenet B. Semont A. (2006). Local irradiation not only induces homing of human Mesenchymal stem cells at exposed sites but promotes their widespread engraftment to multiple organs: a study of their quantitative distribution after irradiation damage. *Stem Cells* 24 1020–1029. 10.1634/stemcells.2005-0260 16339642

[B72] Friedenstein A. J. Chailakhjan R. K. Lalykina K. S. (1970). The development of fibroblast colonies in monolayer cultures of guinea-pig bone marrow and spleen cells. *Cell Prolif* 3, 393–403. 10.1111/j.1365-2184.1970.tb00347.x 5523063

[B73] Friedenstein A. J. Petrakova K. V. Kurolesova A. I. Frolova G. P. (1968). Heterotopic of bone marrow. Analysis of precursor cells for osteogenic and hematopoietic tissues. *Transplantation* 6, 230–247. Available at: https://journals.lww.com/transplantjournal/Abstract/1968/03000/HETEROTOPIC_TRANSPLANTS_OF_BONE_MARROW.9.aspx 5654088

[B74] Galiè M. Konstantinidou G. Peroni D. Scambi I. Marchini C. Lisi V. (2008). Mesenchymal stem cells share molecular signature with mesenchymal tumor cells and favor early tumor growth in syngeneic mice. *Oncogene* 27 2542–2551. 10.1038/sj.onc.1210920 17998939

[B75] Galipeau J. (2013). The mesenchymal stromal cells dilemma- does a negative phase III trial of random donor mesenchymal stromal cells in steroid-resistant graft-versus-host disease represent a death knell or a bump in the road? *Cytotherapy* 15 2–8. 10.1016/j.jcyt.2012.10.002 23260081

[B76] Galipeau J. Sensébé L. (2018). Mesenchymal stromal cells: clinical challenges and therapeutic opportunities. *Cell Stem Cell* 22 824–833. 10.1016/j.stem.2018.05.004 29859173 PMC6434696

[B77] Gao H. Priebe W. Glod J. Banerjee D. (2009). Activation of signal transducers and activators of transcription 3 and focal adhesion kinase by stromal cell-derived factor 1 is required for migration of human mesenchymal stem cells in response to tumor cell-conditioned medium. *Stem Cells* 27 857–865. 10.1002/stem.23 19350687

[B78] García-Olmo D. García-Arranz M. Herreros D. Pascual I. Peiro C. Rodríguez-Montes J. A. (2005). A Phase I clinical trial of the treatment of crohn’s fistula by adipose mesenchymal stem cell transplantation. *Dis. Colon Rectum* 48 1416–1423. 10.1007/s10350-005-0052-6 15933795

[B79] Girdlestone J. (2016). Mesenchymal stromal cells with enhanced therapeutic properties. *Immunotherapy* 8 1405–1416. 10.2217/imt-2016-0098 28000538

[B80] Gong M. Yu B. Wang J. Wang Y. Liu M. Paul C. (2017). Mesenchymal stem cells release exosomes that transfer miRNAs to endothelial cells and promote angiogenesis. *Oncotarget* 8 45200–45212. 10.18632/oncotarget.16778 28423355 PMC5542178

[B81] Grégoire C. Ritacco C. Hannon M. Seidel L. Delens L. Belle L. (2019). Comparison of Mesenchymal stromal cells from different origins for the treatment of graft-vs.-host-disease in a humanized mouse model. *Front. Immunol.* 10:619. 10.3389/fimmu.2019.00619 31001253 PMC6454068

[B82] Guéry J. C. Adorini L. (1995). Dendritic cells are the most efficient in presenting endogenous naturally processed self-epitopes to class II-restricted T cells. *J. Immunol.* 154 536–544. 7529278

[B83] Haddad R. Saldanha-Araujo F. (2014). Mechanisms of T-Cell Immunosuppression by Mesenchymal stromal cells: what do we know so far? *Biomed. Res. Int.* 2014 1–14. 10.1155/2014/216806 25025040 PMC4082893

[B84] Hahn J.-Y. Cho H.-J. Kang H.-J. Kim T.-S. Kim M.-H. Chung J.-H. (2008). Pre-Treatment of mesenchymal stem cells with a combination of growth factors enhances gap junction formation, cytoprotective effect on cardiomyocytes, and therapeutic efficacy for myocardial infarction. *J. Am. Coll. Cardiol.* 51 933–943. 10.1016/J.JACC.2007.11.040 18308163

[B85] Hammerich L. Marron T. U. Upadhyay R. Svensson-Arvelund J. Dhainaut M. Hussein S. (2019). Systemic clinical tumor regressions and potentiation of PD1 blockade with in situ vaccination. *Nat. Med.* 25 814–824. 10.1038/s41591-019-0410-x 30962585

[B86] Han I. Lee M. R. Nam K. W. Oh J. H. Moon K. C. Kim H. S. (2018). Expression of macrophage migration inhibitory factor relates to survival in high-grade osteosarcoma. *Clin. Orthop. Relat. Res.* 466 2107–2113. 10.1007/s11999-008-0333-1 18563508 PMC2492999

[B87] Han Z. Jing Y. Zhang S. Liu Y. Shi Y. Wei L. (2012). The role of immunosuppression of mesenchymal stem cells in tissue repair and tumor growth. *Cell Biosci.* 2:8. 10.1186/2045-3701-2-8 22390479 PMC3315743

[B88] Hanahan D. Weinberg R. A. (2000). The hallmarks of cancer. *Cell* 100 57–70. 10.1016/s0092-8674(00)81683-9 10647931

[B89] Hayashi Y. Tsuji S. Tsujii M. Nishida T. Ishii S. Iijima H. (2008). Topical implantation of Mesenchymal stem cells has beneficial effects on healing of experimental colitis in rats. *J. Pharmacol. Exp. Ther.* 326 523–531. 10.1124/JPET.108.137083 18448866

[B90] Haynesworth S. E. Baber M. A. Caplan A. I. (1996). Cytokine expression by human marrow-derived mesenchymal progenitor cells in vitro: effects of dexamethasone and IL-1α. *J. Cell Physiol.* 166 585–592. 8600162 10.1002/(SICI)1097-4652(199603)166:3<585::AID-JCP13>3.0.CO;2-6

[B91] Higano C. S. Small E. J. Schellhammer P. Yasothan U. Gubernick S. Kirkpatrick P. (2010). Sipuleucel-T. *Nat. Rev. Drug Discov.* 9 513–514. 10.1038/nrd3220 20592741

[B92] Hobernik D. Bros M. (2018). DNA vaccines-how far from clinical use? *Int. J. Mol. Sci.* 19:E3605. 10.3390/ijms19113605 30445702 PMC6274812

[B93] Horwitz E. M. Gordon P. L. Koo W. K. K. Marx J. C. Neel M. D. McNall R. Y. (2002). Isolated allogeneic bone marrow-derived mesenchymal cells engraft and stimulate growth in children with osteogenesis imperfecta: implications for cell therapy of bone. *Proc. Natl. Acad. Sci. U.S.A.* 99 8932–8937. 10.1073/pnas.132252399 12084934 PMC124401

[B94] Horwitz E. M. Prockop D. J. Fitzpatrick L. A. Koo W. W. K. Gordon P. L. Neel M. (1999). Transplantability and therapeutic effects of bone marrow-derived mesenchymal cells in children with osteogenesis imperfecta. *Nat. Med.* 5 309–313. 10.1038/6529 10086387

[B95] Inada M. Follenzi A. Cheng K. Surana M. Joseph B. Benten D. (2008). Phenotype reversion in fetal human liver epithelial cells identifies the role of an intermediate meso-endodermal stage before hepatic maturation. *J. Cell Sci.* 121 1002–1013. 10.1242/jcs.019315 18319302 PMC2695499

[B96] Inoue Y. Iriyama A. Ueno S. Takahashi H. Kondo M. Tamaki Y. (2007). Subretinal transplantation of bone marrow mesenchymal stem cells delays retinal degeneration in the RCS rat model of retinal degeneration. *Exp. Eye Res.* 85 234–241. 10.1016/J.EXER.2007.04.007 17570362

[B97] Introna M. Lucchini G. Dander E. Galimberti S. Rovelli A. Balduzzi A. (2014). Treatment of graft versus host disease with mesenchymal stromal cells: a phase i study on 40 adult and pediatric patients. *Biol. Blood Marrow Transplant.* 20 375–381. 10.1016/j.bbmt.2013.11.033 24321746

[B98] Janikashvili N. Larmonier N. Katsanis E. (2010). Personalized dendritic cell-based tumor immunotherapy. *Immunotherapy* 2 57–68. 10.2217/imt.09.78 20161666 PMC2819192

[B99] Jarosławski S. Toumi M. (2015). Sipuleucel-T (Provenge^®^) - autopsy of an innovative paradigm change in cancer treatment: why a single-product biotech company failed to capitalize on its breakthrough invention. *Biodrugs* 29 301–307. 10.1007/s40259-015-0140-7 26403092

[B100] JCR Pharmaceuticals Co (2015). *TEMCELL^®^ HS Inj.* Available at: https://www.jcrpharm.co.jp/en/site/en/biopharmaceutical/product_tem.html (accessed September 15, 2019).

[B101] Jewett A. Arasteh A. Tseng H.-C. Behel A. Arasteh H. Yang W. (2010). Strategies to rescue Mesenchymal stem cells (MSCs) and dental pulp stem cells (DPSCs) from NK cell mediated cytotoxicity. *PLoS One* 5:e9874. 10.1371/journal.pone.0009874 20360990 PMC2847602

[B102] Joyce J. A. Fearon D. T. (2015). T cell exclusion, immune privilege, and the tumor microenvironment. *Science* 348 74–80. 10.1126/science.aaa6204 25838376

[B103] Karnoub A. E. Dash A. B. Vo A. P. Sullivan A. Brooks M. W. Bell G. W. (2007). Mesenchymal stem cells within tumour stroma promote breast cancer metastasis. *Nature* 449 557–563. 10.1038/nature06188 17914389

[B104] Kean T. J. Lin P. Caplan A. I. Dennis J. E. (2013). MSCs: delivery routes and engraftment, cell-targeting strategies, and immune modulation. *Stem Cells Int.* 2013 732742. 10.1155/2013/732742 24000286 PMC3755386

[B105] Kern S. Eichler H. Stoeve J. Klüter H. Bieback K. (2006). Comparative analysis of mesenchymal stem cells from bone marrow, umbilical cord blood, or adipose tissue. *Stem Cells* 24 1294–1301. 10.1634/stemcells.2005-0342 16410387

[B106] Keyser K. A. Beagles K. E. Kiem H.-P. (2007). Comparison of mesenchymal stem cells from different tissues to suppress t-cell activation. *Cell Transplant.* 16 555–562. 10.3727/000000007783464939 17708345

[B107] Khakoo A. Y. Pati S. Anderson S. A. Reid W. Elshal M. F. Rovira I. I. (2006). Human mesenchymal stem cells exert potent antitumorigenic effects in a model of Kaposi’s sarcoma. *J. Exp. Med.* 203 1235–1247. 10.1084/JEM.20051921 16636132 PMC2121206

[B108] Kharaziha P. Hellström P. M. Noorinayer B. Farzaneh F. Aghajani K. Jafari F. (2009). Improvement of liver function in liver cirrhosis patients after autologous mesenchymal stem cell injection: a phase I–II clinical trial. *Eur. J. Gastroenterol. Hepatol.* 21 1199–1205. 10.1097/MEG.0b013e32832a1f6c 19455046

[B109] Klinge P. M. Harmening K. Miller M. C. Heile A. Wallrapp C. Geigle P. (2011). Encapsulated native and glucagon-like peptide-1 transfected human mesenchymal stem cells in a transgenic mouse model of Alzheimer’s disease. *Neurosci. Lett.* 497 6–10. 10.1016/j.neulet.2011.03.092 21507341

[B110] Klopp A. H. Gupta A. Spaeth E. Andreeff M. Marini F. (2011). Concise review: dissecting a discrepancy in the literature: do mesenchymal stem cells support or suppress tumor growth? *Stem Cells* 29 11–19. 10.1002/stem.559 21280155 PMC3059412

[B111] Komarova S. Roth J. Alvarez R. Curiel D. T. Pereboeva L. (2010). Targeting of mesenchymal stem cells to ovarian tumors via an artificial receptor. *J. Ovarian Res.* 3:12. 10.1186/1757-2215-3-12 20500878 PMC2883983

[B112] Kota D. J. Dicarlo B. Hetz R. A. Smith P. Cox C. S. Olson S. D. (2014). Differential MSC activation leads to distinct mononuclear leukocyte binding mechanisms. *Sci. Rep.* 4:4565. 10.1038/srep04565 24691433 PMC3972508

[B113] Krampera M. (2011). Mesenchymal stromal cell ‘licensing’: a multistep process. *Leukemia* 25 1408–1414. 10.1038/leu.2011.108 21617697

[B114] Krampera M. Cosmi L. Angeli R. Pasini A. Liotta F. Andreini A. (2006). Role for interferon-gamma in the immunomodulatory activity of human bone marrow mesenchymal stem cells. *Stem Cells* 24 386–398. 10.1634/stemcells.2005-0008 16123384

[B115] Krampera M. Glennie S. Dyson J. Scott D. Laylor R. Simpson E. (2003). Bone marrow mesenchymal stem cells inhibit the response of naive and memory antigen-specific T cells to their cognate peptide. *Blood* 101 3722–3729. 10.1182/blood-2002-07-2104 12506037

[B116] Krampera M. Sartoris S. Liotta F. Pasini A. Angeli R. Cosmi L. (2007). Immune regulation by Mesenchymal stem cells derived from adult spleen and thymus. *Stem Cells Dev.* 16 797–810. 10.1089/scd.2007.0024 17999601

[B117] Kubrova E. Qu W. Galvan M. L. Paradise C. R. Yang J. Dietz A. B. (2019). Hypothermia and nutrient deprivation alter viability of human adipose-derived mesenchymal stem cells. *Gene* 722:144058. 10.1016/j.gene.2019.144058 31494240 PMC7309368

[B118] Kuçi Z. Bönig H. Kreyenberg H. Bunos M. Jauch A. Janssen J. W. G. (2016). Mesenchymal stromal cells from pooled mononuclear cells of multiple bone marrow donors as rescue therapy in pediatric severe steroid-refractory graft-versus-host disease: a multicenter survey. *Haematologica* 101 985–994. 10.3324/haematol.2015.140368 27175026 PMC4967578

[B119] Kucia M. Jankowski K. Reca R. Wysoczynski M. Bandura L. Allendorf D. J. (2004). CXCR4-SDF-1 signalling, locomotion, chemotaxis and adhesion. *J. Mol. Histol.* 35 233–245. 10.1023/B:HIJO.0000032355.66152.b8 15339043

[B120] Kurtzberg J. Prasad V. Grimley M. S. Horn B. Carpenter P. A. Jacobsohn D. (2010). Allogeneic human mesenchymal stem cell therapy (Prochymal^®^) as a rescue agent for severe treatment resistant GVHD in pediatric patients. *Biol. Blood Marrow Transplant.* 20 229–235. 10.1016/j.bbmt.2009.12.056 24216185

[B121] Kuznetsov S. A. Krebsbach P. H. Satomura K. Kerr J. Riminucci M. Benayahu D. (1997). Single-colony derived strains of human marrow stromal fibroblasts form bone after transplantation in vivo. *J. Bone Miner. Res.* 12 1335–1347. 10.1359/jbmr.1997.12.9.1335 9286749

[B122] Kyriakidis T. Iosifidis M. Michalopoulos E. Melas I. Stavropoulos-Giokas C. Verdonk R. (2019). Good mid-term outcomes after adipose-derived culture-expanded mesenchymal stem cells implantation in knee focal cartilage defects. *Knee Sur. Sport Traumatol. Arthrosc.* 10.1007/s00167-019-05688-9 [Epub ahead of print]. 31493012

[B123] Kyriakou C. Rabin N. Pizzey A. Nathwani A. Yong K. (2008). Factors that influence short-term homing of human bone marrow-derived mesenchymal stem cells in a xenogeneic animal model. *Haematologica* 93 1457–1465. 10.3324/haematol.12553 18728032

[B124] Law S. Chaudhuri S. (2013). Mesenchymal stem cell and regenerative medicine: regeneration versus immunomodulatory challenges. *Am. J. Stem Cells* 2 22–38. 23671814 PMC3636724

[B125] Le Blanc K. Frassoni F. Ball L. Locatelli F. Roelofs H. Lewis I. (2008). Mesenchymal stem cells for treatment of steroid-resistant, severe, acute graft-versus-host disease: a phase II study. *Lancet* 371 1579–1586. 10.1016/S0140-6736(08)60690-X 18468541

[B126] Le Blanc K. Mougiakakos D. (2012). Multipotent mesenchymal stromal cells and the innate immune system. *Nat. Rev. Immunol.* 12 383–396. 10.1038/nri3209 22531326

[B127] Le Blanc K. Rasmusson I. Sundberg B. Götherström C. Hassan M. Uzunel M. (2004). Treatment of severe acute graft-versus-host disease with third party haploidentical mesenchymal stem cells. *Lancet* 363 1439–1441. 10.1016/S0140-6736(04)16104-7 15121408

[B128] Le D. T. Pardoll D. M. Jaffee E. M. (2010). Cellular vaccine approaches. *Cancer J.* 16 304–310. 10.1097/PPO.0b013e3181eb33d7 20693840 PMC3086689

[B129] Lechanteur C. Briquet A. Giet O. Delloye O. Baudoux E. Beguin Y. (2016). Clinical-scale expansion of mesenchymal stromal cells: a large banking experience. *J. Transl. Med.* 14:145. 10.1186/s12967-016-0892-y 27207011 PMC4875672

[B130] Lee J. W. Fang X. Gupta N. Serikov V. Matthay M. A. (2009). Allogeneic human mesenchymal stem cells for treatment of E. coli endotoxin-induced acute lung injury in the ex vivo perfused human lung. *Proc. Natl. Acad. Sci. U.S.A.* 106 16357–16362. 10.1073/pnas.0907996106 19721001 PMC2735560

[B131] Lee S. J. Vogelsang G. Flowers M. E. (2003). Chronic graft-versus-host disease. *Biol. Blood Marrow Transplant.* 9 215–233. 10.1053/bbmt.2003.50026 12720215

[B132] Levy O. Brennen W. N. Han E. Rosen D. M. Musabeyezu J. Safaee H. (2016). A prodrug-doped cellular Trojan Horse for the potential treatment of prostate cancer. *Biomaterials* 91 140–150. 10.1016/j.biomaterials.2016.03.023 27019026 PMC4824400

[B133] Li H. Yu B. Zhang Y. Pan Z. Xu W. Li H. (2006). Jagged1 protein enhances the differentiation of mesenchymal stem cells into cardiomyocytes. *Biochem. Biophys. Res. Commun.* 341 320–325. 10.1016/J.BBRC.2005.12.182 16413496

[B134] Li X. Lu Y. Huang W. Xu H. Chen X. Geng Q. (2006). In vitro effect of adenovirus-mediated human gamma interferon gene transfer into human mesenchymal stem cells for chronic myelogenous leukemia. *Hematol. Oncol.* 24 151–158. 10.1002/hon.779 16700092

[B135] Lin W. Huang L. Li Y. Fang B. Li G. Chen L. (2019). Mesenchymal stem cells and cancer: clinical challenges and opportunities. *Biomed. Res. Int.* 2019 1–12. 10.1155/2019/2820853 31205939 PMC6530243

[B136] Liotta F. Angeli R. Cosmi L. Filì L. Manuelli C. Frosali F. (2008). Toll-like receptors 3 and 4 are expressed by human bone marrow-derived Mesenchymal stem cells and can inhibit their t-cell modulatory activity by impairing notch signaling. *Stem Cells* 26 279–289. 10.1634/stemcells.2007-0454 17962701

[B137] Liu H. Li D. Zhang Y. Li M. (2018). Inflammation, mesenchymal stem cells and bone regeneration. *Histochem. Cell. Biol.* 149 393–404. 10.1007/s00418-018-1643-3 29435765

[B138] Llevadot J. Murasawa S. Kureishi Y. Uchida S. Masuda H. Kawamoto A. (2001). HMG-CoA reductase inhibitor mobilizes bone marrow-derived endothelial progenitor cells. *J. Clin. Invest.* 108 399–405. 10.1172/JCI13131 11489933 PMC209363

[B139] Locatelli F. Algeri M. Trevisan V. Bertaina A. (2017). Remestemcel-L for the treatment of graft versus host disease. *Expert. Rev. Clin. Immunol.* 13 43–56. 10.1080/1744666X.2016.1208086 27399600

[B140] Loebinger M. R. Eddaoudi A. Davies D. Janes S. M. (2009). Mesenchymal stem cell delivery of TRAIL can eliminate metastatic cancer. *Cancer Res.* 69 4134–4142. 10.1158/0008-5472.CAN-08-4698 19435900 PMC2699841

[B141] Loebinger M. R. Janes S. M. (2010). Stem cells as vectors for antitumour therapy. *Thorax* 65 362–369. 10.1136/thx.2009.128025 20388765 PMC3401681

[B142] Lourenco S. Teixeira V. H. Kalber T. Jose R. J. Floto R. A. Janes S. M. (2015). Macrophage migration inhibitory factor–CXCR4 Is the dominant chemotactic axis in human mesenchymal stem cell recruitment to tumors. *J. Immunol.* 194 3463–3474. 10.4049/jimmunol.1402097 25712213 PMC4374168

[B143] Lu D. Chen B. Liang Z. Deng W. Jiang Y. Li S. (2011). Comparison of bone marrow mesenchymal stem cells with bone marrow-derived mononuclear cells for treatment of diabetic critical limb ischemia and foot ulcer: a double-blind, randomized, controlled trial. *Diabetes Res. Clin. Pract.* 92 26–36. 10.1016/j.diabres.2010.12.010 21216483

[B144] Lu Y. Yuan Y. Wang X. Wei L. Chen Y. Cong C. (2008). The growth inhibitory effect of mesenchymal stem cells on tumor cells in vitro and in vivo. *Cancer Biol. Ther.* 7 245–251. 10.4161/cbt.7.2.5296 18059192

[B145] Lucarelli E. Beccheroni A. Donati D. Sangiorgi L. Cenacchi A. Del Vento A. M. (2003). Platelet-derived growth factors enhance proliferation of human stromal stem cells. *Biomaterials* 24 3095–3100. 10.1016/S0142-9612(03)00114-5 12895582

[B146] Luft T. Dietrich S. Falk C. Conzelmann M. Hess M. Benner A. (2011). Steroid-refractory GVHD: T-cell attack within a vulnerable endothelial system. *Blood* 118 1685–1692. 10.1182/blood-2011-02-334821 21636856

[B147] Lüttichau I. Von Notohamiprodjo M. Wechselberger A. Peters C. Henger A. Seliger C. (2005). Human adult CD34 - progenitor cells functionally express the chemokine receptors CCR1, CCR4, CCR7, CXCR5, and CCR10 but Not CXCR4. *Stem Cells Dev.* 14 329–336. 10.1089/scd.2005.14.329 15969628

[B148] Ma T. Gong K. Ao Q. Yan Y. Song B. Huang H. (2013). Intracerebral transplantation of adipose-derived mesenchymal stem cells alternatively activates microglia and ameliorates neuropathological deficits in alzheimer’s disease mice. *Cell Transplant.* 22 113–126. 10.3727/096368913X672181 24070198

[B149] Mabuchi Y. Houlihan D. D. Okano H. Matsuzaki Y. (2012). Discovering the true identity and function of mesenchymal stem cells. *Inflamm. Regen.* 32 146–151. 10.2492/inflammregen.32.146

[B150] MacGregor R. R. Boyer J. D. Ugen K. E. Lacy K. E. Gluckman S. J. Bagarazzi M. L. (1998). First human trial of a DNA-based vaccine for treatment of human immunodeficiency virus type 1 infection: safety and host response. *J. Infect. Dis.* 178 92–100. 10.1086/515613 9652427

[B151] Maestroni G. J. M. Hertens E. Galli P. (1999). Factor(s) from nonmacrophage bone marrow stromal cells inhibit Lewis lung carcinoma and B16 melanoma growth in mice. *Cell Mol. Life Sci.* 55 663–667. 10.1007/s000180050322 10357234 PMC11147120

[B152] Majumdar M. K. Keane-Moore M. Buyaner D. Hardy W. B. Moorman M. A. McIntosh K. R. (2003). Characterization and functionality of cell surface molecules on human mesenchymal stem cells. *J. Biomed. Sci.* 10 228–241. 10.1007/bf02256058 12595759

[B153] Mantia-Smaldone G. M. Chu C. S. (2013). A review of dendritic cell therapy for cancer: progress and challenges. *Biodrugs* 27 453–468. 10.1007/s40259-013-0030-9 23592406

[B154] Marks P. W. Witten C. M. Califf R. M. (2017). Clarifying stem-cell therapy’s benefits and risks. *N. Engl. J. Med.* 376 1007–1009. 10.1056/NEJMp1613723 27959704

[B155] Martin P. J. Uberti J. P. Soiffer R. J. Klingemann H. Waller E. K. Daly A. S. (2010). Prochymal improves response rates in patients with steroid-refractory acute graft versus host disease (SR-GVHD) involving the liver and gut: results of a randomized, placebo-controlled, multicenter phase III trial in GVHD. *Biol. Blood Marrow Transplant.* 16 S169–S170. 10.1016/j.bbmt.2009.12.057

[B156] Mellman I. Steinman R. M. (2001). Dendritic cells: specialized and regulated antigen processing machines. *Cell* 106 255–258. 11509172 10.1016/s0092-8674(01)00449-4

[B157] Méndez-Ferrer S. Michurina T. V. Ferraro F. Mazloom A. R. MacArthur B. D. Lira S. A. (2010). Mesenchymal and haematopoietic stem cells form a unique bone marrow niche. *Nature* 466 829–834. 10.1038/nature09262 20703299 PMC3146551

[B158] MilliporeSigma (2017). *Renaissance in Immunotherapy in South Korea.* Available at: https://www.emdmillipore.com/INTERSHOP/static/WFS/Merck-Site/-/Merck/en_US/EmergingBiotech/downloads/PR1254ENUS.pdf (accessed October 21, 2019).

[B159] Min J.-Y. Sullivan M. F. Yang Y. Zhang J.-P. Converso K. L. Morgan J. P. (2002). Significant improvement of heart function by cotransplantation of human mesenchymal stem cells and fetal cardiomyocytes in postinfarcted pigs. *Ann. Thorac. Surg.* 74 1568–1575. 10.1016/s0003-4975(02)03952-8 12440610

[B160] Mohyeldin A. Garzón-Muvdi T. Quiñones-Hinojosa A. (2010). Oxygen in stem cell biology: a critical component of the stem cell niche. *Cell Stem Cell* 7 150–161. 10.1016/j.stem.2010.07.007 20682444

[B161] Murphy J. M. Dixon K. Beck S. Fabian D. Feldman A. Barry F. (2002). Reduced chondrogenic and adipogenic activity of mesenchymal stem cells from patients with advanced osteoarthritis. *Arthrit. Rheum.* 46 704–713. 10.1002/art.10118 11920406

[B162] Nakamura K. Ito Y. Kawano Y. Kurozumi K. Kobune M. Tsuda H. (2004). Antitumor effect of genetically engineered mesenchymal stem cells in a rat glioma model. *Gene Ther.* 11 1155–1164. 10.1038/sj.gt.3302276 15141157

[B163] Nayereh K. G. Khadem G. (2012). Preventive and therapeutic vaccines against human papillomaviruses associated cervical cancers. *Iran J. Basic Med. Sci.* 15 585–601. 23493151 PMC3586871

[B164] Németh K. Leelahavanichkul A. Yuen P. S. T. Mayer B. Parmelee A. Doi K. (2009). Bone marrow stromal cells attenuate sepsis via prostaglandin E2–dependent reprogramming of host macrophages to increase their interleukin-10 production. *Nat. Med.* 15 42–49. 10.1038/nm.1905 19098906 PMC2706487

[B165] Nicolette C. A. Healey D. Tcherepanova I. Whelton P. Monesmith T. Coombs L. (2007). Dendritic cells for active immunotherapy: optimizing design and manufacture in order to develop commercially and clinically viable products. *Vaccine* 25 B47–B60. 10.1016/j.vaccine.2007.06.006 17669561

[B166] Niess H. von Einem J. C. Thomas M. N. Michl M. Angele M. K. Huss R. (2015). Treatment of advanced gastrointestinal tumors with genetically modified autologous mesenchymal stromal cells (TREAT-ME1): study protocol of a phase I/II clinical trial. *BMC Cancer* 15:237. 10.1186/s12885-015-1241-x 25879229 PMC4393860

[B167] NIH (2019). *ClinicalTrials.gov.* Available at: https://clinicaltrials.gov/ct2/home (accessed September 15, 2019).

[B168] Ohgushi H. Kotobuki N. Funaoka H. Machida H. Hirose M. Tanaka Y. (2005). Tissue engineered ceramic artificial joint–ex vivo osteogenic differentiation of patient mesenchymal cells on total ankle joints for treatment of osteoarthritis. *Biomaterials* 26 4654–4661. 10.1016/j.biomaterials.2004.11.055 15722135

[B169] Orthocell (2017). *Ortho-ACI^TM^ Consumer Medicines Information.* Available at: https://static1.squarespace.com/static/55d2ae4ce4b0e20eb51007ce/t/59a6e368ccc5c51f72dc245b/1504109419480/10-IFU-10+Ortho-ACI.+Consumer+Medicines+Information+v2.pdf (accessed September 15, 2019).

[B170] Otsu K. Das S. Houser S. D. Quadri S. K. Bhattacharya S. Bhattacharya J. (2009). Concentration-dependent inhibition of angiogenesis by mesenchymal stem cells. *Blood* 113 4197–4205. 10.1182/blood-2008-09-176198 19036701 PMC2676081

[B171] Ott P. A. Hu Z. Keskin D. B. Shukla S. A. Sun J. Bozym D. J. (2017). An immunogenic personal neoantigen vaccine for patients with melanoma. *Nature* 547 217–221. 10.1038/nature22991 28678778 PMC5577644

[B172] Ouyang H. W. Goh J. C. H. Thambyah A. Teoh S. H. Lee E. H. (2003). Knitted poly-lactide-co-glycolide scaffold loaded with bone marrow stromal cells in repair and regeneration of rabbit achilles tendon. *Tissue Eng.* 9 431–439. 10.1089/107632703322066615 12857411

[B173] Palucka K. Banchereau J. (2013). Dendritic-cell-based therapeutic cancer vaccines. *Immunity* 39 38–48. 10.1016/j.immuni.2013.07.004 23890062 PMC3788678

[B174] Panés J. García-Olmo D. Van Assche G. Colombel J. F. Reinisch W. Baumgart D. C. (2016). Expanded allogeneic adipose-derived mesenchymal stem cells (Cx601) for complex perianal fistulas in Crohn’s disease: a phase 3 randomised, double-blind controlled trial. *Lancet* 388 1281–1290. 10.1016/S0140-6736(16)31203-X 27477896

[B175] Panés J. García-Olmo D. Van Assche G. Colombel J. F. Reinisch W. Baumgart D. C. (2018). Long-term efficacy and safety of stem cell therapy (Cx601) for complex perianal fistulas in patients with Crohn’s Disease. *Gastroenterology* 154 1334.e–1342.e. 10.1053/j.gastro.2017.12.020 29277560

[B176] Panes J. Garcia-Olmo D. Van Assche G. A. Colombel J. F. Reinisch W. Baumgart D. C. (2017). CX601, allogeneic expanded adipose-derived mesenchymal stem cells (EASC), for complex perianal fistulas in crohn’s disease: long-term results from a phase iii randomized controlled trial. *Gastroenterology* 15:S187 10.1016/s0016-5085(17)30934-4 27477896

[B177] Patel S. A. Meyer J. R. Greco S. J. Corcoran K. E. Bryan M. Rameshwar P. (2010). Mesenchymal stem cells protect breast cancer cells through regulatory T cells: role of mesenchymal stem cell-derived TGF-beta. *J. Immunol.* 184 5885–5894. 10.4049/jimmunol.0903143 20382885

[B178] Pavlova G. Lopatina T. Kalinina N. Rybalkina E. Parfyonova Y. Tkachuk V. (2012). In vitro neuronal induction of adipose-derived stem cells and their fate after transplantation into injured mouse brain. *Curr. Med. Chem.* 19 5170–5177. 10.2174/092986712803530557 22934763

[B179] Peng L. Xie D. Lin B.-L. Liu J. Zhu H. Xie C. (2011). Autologous bone marrow mesenchymal stem cell transplantation in liver failure patients caused by hepatitis B: short-term and long-term outcomes. *Hepatology* 54 820–828. 10.1002/hep.24434 21608000

[B180] Penn M. S. Khalil M. K. (2008). Exploitation of stem cell homing for gene delivery. *Expert. Opin. Biol. Ther.* 8 17–30. 10.1517/14712598.8.1.17 18081534

[B181] Pessina A. Bonomi A. Coccè V. Invernici G. Navone S. Cavicchini L. (2011). Mesenchymal stromal cells primed with paclitaxel provide a new approach for cancer therapy. *PLoS One* 6:e28321. 10.1371/journal.pone.0028321 22205945 PMC3243689

[B182] Peters B. A. Diaz L. A. Polyak K. Meszler L. Romans K. Guinan E. C. (2005). Contribution of bone marrow–derived endothelial cells to human tumor vasculature. *Nat. Med.* 11 261–262. 10.1038/nm1200 15723071

[B183] PHARMICELL, (2011). *Cellgram^®^-AMI.* Available at: http://www.pharmicell.com/eng/biz/medicine_cellgram.html# (accessed September 15, 2019).

[B184] Phillips R. J. Burdick M. D. Lutz M. Belperio J. A. Keane M. P. Strieter R. M. (2003). The stromal derived factor-1/CXCL12-CXC chemokine receptor 4 biological axis in non-small cell lung cancer metastases. *Am. J. Respir. Crit. Care Med.* 167 1676–1686. 10.1164/rccm.200301-071OC 12626353

[B185] Phinney D. G. Pittenger M. F. (2017). Concise review: msc-derived exosomes for cell-free therapy. *Stem Cells* 35 851–858. 10.1002/stem.2575 28294454

[B186] Polchert D. Sobinsky J. Douglas G. Kidd M. Moadsiri A. Reina E. (2008). IFN-γ activation of mesenchymal stem cells for treatment and prevention of graft versus host disease. *Eur. J. Immunol.* 38 1745–1755. 10.1002/eji.200738129 18493986 PMC3021120

[B187] Poncelet A. J. Vercruysse J. Saliez A. Gianello P. (2007). Although pig allogeneic mesenchymal stem cells are not immunogenic in vitro, intracardiac injection elicits an immune response in vivo. *Transplantation* 2017:9824698. 10.1097/01.tp.0000258649.23081.a3 17414713

[B188] Prockop D. J. (1997). Marrow stromal cells as stem cells for nonhematopoietic tissues. *Science* 276 71–74. 10.1126/science.276.5309.71 9082988

[B189] Prockop D. J. Kota D. J. Bazhanov N. Reger R. L. (2010). Evolving paradigms for repair of tissues by adult stem/progenitor cells (MSCs). *J. Cell. Mol. Med.* 14 2190–2199. 10.1111/j.1582-4934.2010.01151.x 20716123 PMC3489272

[B190] Qiao L. Xu Z. Zhao T. Ye L. Zhang X. (2008). Dkk-1 secreted by mesenchymal stem cells inhibits growth of breast cancer cells via depression of Wnt signalling. *Cancer Lett.* 269 67–77. 10.1016/J.CANLET.2008.04.032 18571836

[B191] Rafei M. Campeau P. M. Aguilar-Mahecha A. Buchanan M. Williams P. Birman E. (2009). Mesenchymal Stromal cells ameliorate experimental autoimmune encephalomyelitis by inhibiting cd4 th17 t cells in a cc chemokine ligand 2-dependent manner. *J. Immunol.* 182 5994–6002. 10.4049/jimmunol.0803962 19414750

[B192] Rafei M. Hsieh J. Fortier S. Li M. Yuan S. Birman E. (2008). Mesenchymal stromal cell–derived CCL2 suppresses plasma cell immunoglobulin production via STAT3 inactivation and PAX5 induction. *Blood* 112 4991–4998. 10.1182/blood-2008-07-166892 18812467

[B193] Rasmusson I. Le Blanc K. Sundberg B. Ringdén O. (2007). Mesenchymal stem cells stimulate antibody secretion in human B cells. *Scand. J. Immunol.* 65 336–343. 10.1111/j.1365-3083.2007.01905.x 17386024

[B194] Rasulov M. F. Vasil’chenkov A. V. Onishchenko N. A. Krasheninnikov M. E. Kravchenko V. I. Gorshenin T. L. (2005). First experience in the use of bone marrow mesenchymal stem cells for the treatment of a patient with deep skin burns. *Bull. Exp. Biol. Med.* 139 141–144. 10.1007/s10517-005-0232-3 16142297

[B195] Reagan M. R. Kaplan D. L. (2011). Mesenchymal stem cell tumor-homing: detection methods in disease model systems. *Stem Cells* 29 920–927. 10.1002/stem.645 21557390 PMC4581846

[B196] Redondo L. M. García V. Peral B. Verrier A. Becerra J. Sánchez A. (2018). Repair of maxillary cystic bone defects with mesenchymal stem cells seeded on a cross-linked serum scaffold. *J. Craniomaxillofacial Surg.* 46 222–229. 10.1016/j.jcms.2017.11.004 29229365

[B197] Regrow Biosciences^®^ (2019). *OSSGROW^TM^ for Avascular Necrosis*. Available at: https://www.regrow.in/ossgrow-for-avn (accessed February 3, 2020).

[B198] Ren C. Kumar S. Chanda D. Chen J. Mountz J. D. Ponnazhagan S. (2008a). Therapeutic potential of mesenchymal stem cells producing interferon-α in a mouse melanoma lung metastasis model. *Stem Cells* 26 2332–2338. 10.1634/stemcells.2008-0084 18617688 PMC2940717

[B199] Ren C. Kumar S. Chanda D. Kallman L. Chen J. Mountz J. D. (2008b). Cancer gene therapy using mesenchymal stem cells expressing interferon-β in a mouse prostate cancer lung metastasis model. *Gene Ther.* 15 1446–1453. 10.1038/gt.2008.101 18596829 PMC2766853

[B200] Renner P. Eggenhofer E. Rosenauer A. Popp F. C. Steinmann J. F. Slowik P. (2009). Mesenchymal stem cells require a sufficient, ongoing immune response to exert their immunosuppressive function. *Transplant. Proc.* 41 2607–2611. 10.1016/j.transproceed.2009.06.119 19715984

[B201] Reza A. M. M. T. Choi Y.-J. Yasuda H. Kim J.-H. (2016). Human adipose mesenchymal stem cell-derived exosomal-miRNAs are critical factors for inducing anti-proliferation signalling to A2780 and SKOV-3 ovarian cancer cells. *Sci. Rep.* 6:38498. 10.1038/srep38498 27929108 PMC5143979

[B202] Rigo A. Gottardi M. Zamò A. Mauri P. Bonifacio M. Krampera M. (2010). Macrophages may promote cancer growth via a GM-CSF/HB-EGF paracrine loop that is enhanced by CXCL12. *Mol. Cancer* 9:273. 10.1186/1476-4598-9-273 20946648 PMC2964621

[B203] Rivera-Cruz C. M. Shearer J. J. Neto F. Figueiredo M. L. (2017). The immunomodulatory effects of mesenchymal stem cell polarization within the tumor microenvironment niche. *Stem Cells Int.* 2017:4015039. 10.1155/2017/4015039 29181035 PMC5664329

[B204] Robson N. C. Hoves S. Maraskovsky E. Schnurr M. (2010). Presentation of tumour antigens by dendritic cells and challenges faced. *Curr. Opin. Immunol.* 22 137–144. 10.1016/j.coi.2010.01.002 20116984

[B205] Roccaro A. M. Sacco A. Maiso P. Azab A. K. Tai Y.-T. Reagan M. (2013). BM mesenchymal stromal cell–derived exosomes facilitate multiple myeloma progression. *J. Clin. Invest.* 123 1542–1555. 10.1172/JCI66517 23454749 PMC3613927

[B206] Roddy G. W. Oh J. Y. Lee R. H. Bartosh T. J. Ylostalo J. Coble K. (2011). Action at a distance: systemically administered adult stem/progenitor cells (MSCs) reduce inflammatory damage to the cornea without engraftment and primarily by secretion of TNF-α stimulated gene/protein 6. *Stem Cells* 29 1572–1579. 10.1002/stem.708 21837654

[B207] Romieu-Mourez R. François M. Boivin M.-N. Bouchentouf M. Spaner D. E. Galipeau J. (2009). Cytokine modulation of TLR expression and activation in Mesenchymal stromal cells leads to a proinflammatory phenotype. *J. Immunol.* 182 7963–7973. 10.4049/jimmunol.0803864 19494321

[B208] Russell A. L. Lefavor R. Durand N. Glover L. Zubair A. C. (2018). Modifiers of mesenchymal stem cell quantity and quality. *Transfusion* 58 1434–1440. 10.1111/trf.14597 29582436

[B209] Sasaki M. Abe R. Fujita Y. Ando S. Inokuma D. Shimizu H. (2008). Mesenchymal stem cells are recruited into wounded skin and contribute to wound repair by transdifferentiation into multiple skin cell type. *J. Immunol.* 180 2581–2587. 10.4049/jimmunol.180.4.2581 18250469

[B210] Schumacher T. N. Schreiber R. D. (2015). Neoantigens in cancer immunotherapy. *Science* 348 69–74. 10.1126/science.aaa4971 25838375

[B211] Schweizer M. T. Wang H. Bivalacqua T. J. Partin A. W. Lim S. J. Chapman C. (2019). A phase I study to assess the safety and cancer-homing ability of allogeneic bone marrow-derived Mesenchymal stem cells in men with localized prostate cancer. *Stem Cells Transl. Med.* 8 441–449. 10.1002/sctm.18-0230 30735000 PMC6477003

[B212] Secchiero P. Zorzet S. Tripodo C. Corallini F. Melloni E. Caruso L. (2010). Human bone marrow Mesenchymal stem cells display anti-cancer activity in SCID mice bearing disseminated non-hodgkin’s lymphoma xenografts. *PLoS One* 5:e11140. 10.1371/journal.pone.0011140 20585401 PMC2886845

[B213] Sekiya I. Larson B. L. Smith J. R. Pochampally R. Cui J. Prockop D. J. (2002). Expansion of human adult stem cells from bone marrow stroma: conditions that maximize the yields of early progenitors and evaluate their quality. *Stem Cells* 20 530–541. 10.1634/stemcells.20-6-530 12456961

[B214] Sensebé L. (2008). Clinical grade production of mesenchymal stem cells. *Biomed. Mater. Eng.* 18 S3–S10. 18334718

[B215] Seo S. H. Kim K. S. Park S. H. Suh Y. S. Kim S. J. Jeun S.-S. (2011). The effects of mesenchymal stem cells injected via different routes on modified IL-12-mediated antitumor activity. *Gene Ther.* 18 488–495. 10.1038/gt.2010.170 21228885 PMC3125103

[B216] Serakinci N. Cagsin H. (2019). Turning stem cells homing potential into cancer specific drug delivery machines. *Stem Cells Transl. Med.* 7 441–450. 10.21037/atm.2019.06.30 31576355 PMC6685889

[B217] Sheng G. (2015). The developmental basis of mesenchymal stem/stromal cells (MSCs). *BMC Dev. Biol.* 15:44. 10.1186/s12861-015-0094-5 26589542 PMC4654913

[B218] Sheng H. Wang Y. Jin Y. Zhang Q. Zhang Y. Wang L. (2008). A critical role of IFNγ in priming MSC-mediated suppression of T cell proliferation through up-regulation of B7-H1. *Cell Res.* 18 846–857. 10.1038/cr.2008.80 18607390

[B219] Shi M. Li J. Liao L. Chen B. Li B. Chen L. (2007). Regulation of CXCR4 expression in human mesenchymal stem cells by cytokine treatment: role in homing efficiency in NOD/SCID mice. *Haematologica* 92 897–904. 10.3324/haematol.10669 17606439

[B220] Siegel G. Schäfer R. Dazzi F. (2009). The immunosuppressive properties of Mesenchymal stem cells. *Transplantation* 87 S45–S49. 10.1097/TP.0b013e3181a285b0 19424005

[B221] Singer N. G. Caplan A. I. (2011). Mesenchymal stem cells: mechanisms of inflammation. *Annu. Rev. Pathol. Mech. Dis.* 6 457–478. 10.1146/annurev-pathol-011110-130230 21073342

[B222] Singh A. Singh A. Sen D. (2016). Mesenchymal stem cells in cardiac regeneration: a detailed progress report of the last 6 years (2010-2015). *Stem Cell Res. Ther.* 7:82. 10.1186/s13287-016-0341-0 27259550 PMC4893234

[B223] Sipp D. (2015). Conditional approval: japan lowers the bar for regenerative medicine products. *Cell Stem Cell* 16 353–356. 10.1016/j.stem.2015.03.013 25842975

[B224] Small E. J. Schellhammer P. F. Higano C. S. Redfern C. H. Nemunaitis J. J. Valone F. H. (2006). Placebo-controlled phase III trial of immunologic therapy with Sipuleucel-T (APC8015) in patients with metastatic, asymptomatic hormone refractory prostate cancer. *J. Clin. Oncol.* 24 3089–3094. 10.1200/JCO.2005.04.5252 16809734

[B225] Socié G. Ritz J. (2014). Current issues in chronic graft-versus-host disease. *Blood* 124 374–384. 10.1182/blood-2014-01-514752 24914139 PMC4102710

[B226] Solchaga L. A. Penick K. Porter J. D. Goldberg V. M. Caplan A. I. Welter J. F. (2005). FGF-2 enhances the mitotic and chondrogenic potentials of human adult bone marrow-derived mesenchymal stem cells. *J. Cell Physiol.* 203 398–409. 10.1002/jcp.20238 15521064

[B227] Sotiropoulou P. A. Perez S. A. Gritzapis A. D. Baxevanis C. N. Papamichail M. (2006). Interactions between human mesenchymal stem cells and natural killer cells. *Stem Cells* 24 74–85. 10.1634/stemcells.2004-0359 16099998

[B228] Spaggiari G. M. Moretta L. (2012). “Mesenchymal stem cell-natural killer cell interactions,” in *Stem Cells and Cancer Stem Cells, Volume 4: Therapeutic Applications in Disease and Injury*, ed. Hayat M. A. (Berlin: Springer), 217–224. 10.1007/978-94-007-2828-8-19

[B229] Squillaro T. Peluso G. Galderisi U. (2016). Clinical trials with mesenchymal stem cells: an update. *Cell Transplant.* 25 829–848. 10.3727/096368915X689622 26423725

[B230] Stagg J. Galipeau J. (2013). Mechanisms of immune modulation by Mesenchymal stromal cells and clinical translation. *Curr. Mol. Med.* 13 856–867. 10.2174/1566524011313050016 23642066

[B231] Stagg J. Pommey S. Eliopoulos N. Galipeau J. (2006). Interferon-gamma-stimulated marrow stromal cells: a new type of nonhematopoietic antigen-presenting cells. *Blood* 107 2570–2577. 10.1182/blood-2005-07-2793 16293599

[B232] Steinman R. M. (2001). Dendritic cells and the control of immunity: enhancing the efficiency of antigen presentation. *Mt Sinai J. Med.* 68 160–166. 11373688

[B233] Strioga M. Viswanathan S. Darinskas A. Slaby O. Michalek J. (2012). Same or not the same? comparison of adipose tissue-derived versus bone marrow-derived mesenchymal stem and stromal cells. *Stem Cells Dev.* 21 2724–2752. 10.1089/scd.2011.0722 22468918

[B234] Studeny M. Marini F. C. Champlin R. E. Zompetta C. Fidler I. J. Andreeff M. (2002). Bone marrow-derived Mesenchymal stem cells as vehicles for interferon-beta delivery into tumors. *Cancer Res.* 62 3603–3608. 12097260

[B235] Sudres M. Norol F. Trenado A. Grégoire S. Charlotte F. Levacher B. (2006). Bone marrow Mesenchymal stem cells suppress lymphocyte proliferation in vitro but fail to prevent graft-versus-host disease in mice. *J. Immunol.* 176 7761–7767. 10.4049/jimmunol.176.12.7761 16751424

[B236] Takahashi N. Udagawa N. Suda T. (1999). A new member of tumor necrosis factor ligand family, ODF/OPGL/TRANCE/RANKL, regulates osteoclast differentiation and function. *Biochem. Biophys. Res. Commun.* 256 449–455. 10.1006/BBRC.1999.0252 10080918

[B237] Tappenbeck N. Schröder H. M. Niebergall-Roth E. Hassinger F. Dehio U. Dieter K. (2019). In vivo safety profile and biodistribution of GMP-manufactured human skin-derived ABCB5-positive mesenchymal stromal cells for use in clinical trials. *Cytotherapy* 21 546–560. 10.1016/j.jcyt.2018.12.005 30878384 PMC6513723

[B238] Tisato V. Naresh K. Girdlestone J. Navarrete C. Dazzi F. (2007). Mesenchymal stem cells of cord blood origin are effective at preventing but not treating graft-versus-host disease. *Leukemia* 21 1992–1999. 10.1038/sj.leu.2404847 17625609

[B239] Togel F. Hu Z. Weiss K. Isaac J. Lange C. Westenfelder C. (2005). Amelioration of acute renal failure by stem cell therapy–paracrine secretion Versus transdifferentiation into resident cells. *J. Am. Soc. Nephrol.* 16 1153–1163. 10.1681/ASN.2005030294

[B240] Tögel F. Hu Z. Weiss K. Isaac J. Lange C. Westenfelder C. (2005). Administered mesenchymal stem cells protect against ischemic acute renal failure through differentiation-independent mechanisms. *Am. J. Physiol. Physiol.* 289 F31–F42. 10.1152/ajprenal.00007.2005 15713913

[B241] Tomchuck S. L. Norton E. B. Garry R. F. Bunnell B. A. Morris C. A. Freytag L. C. (2012). Mesenchymal stem cells as a novel vaccine platform. *Front. Cell Infect. Microbiol.* 2:140. 10.3389/fcimb.2012.00140 23162801 PMC3499769

[B242] Tomchuck S. L. Zwezdaryk K. J. Coffelt S. B. Waterman R. S. Danka E. S. Scandurro A. B. (2008). Toll-like receptors on human mesenchymal stem cells drive their migration and immunomodulating responses. *Stem Cells* 26 99–107. 10.1634/stemcells.2007-0563 17916800 PMC2757778

[B243] Tontonoz P. Hu E. Spiegelman B. M. (1994). Stimulation of adipogenesis in fibroblasts by PPARγ2, a lipid-activated transcription factor. *Cell* 79 1147–1156. 10.1016/0092-8674(94)90006-X 8001151

[B244] Tsai P.-J. Yeh C.-C. Huang W.-J. Min M.-Y. Huang T.-H. Ko T.-L. (2019). Xenografting of human umbilical mesenchymal stem cells from Wharton’s jelly ameliorates mouse spinocerebellar ataxia type 1. *Transl. Neurodegener.* 8:29. 10.1186/s40035-019-0166-8 31508229 PMC6727337

[B245] U. S. National Library of Medicine (2019). *Mesenchymal Stem Cells: ClinicalTrials.Gov.* Bethesda, MD: U. S. National Library of Medicine.

[B246] Uchibori R. Okada T. Ito T. Urabe M. Mizukami H. Kume A. (2009). Retroviral vector-producing mesenchymal stem cells for targeted suicide cancer gene therapy. *J. Gene Med.* 11 373–381. 10.1002/jgm.1313 19274675

[B247] Ullah I. Subbarao R. B. Rho G. J. (2015). Human mesenchymal stem cells - current trends and future prospective. *Biosci. Rep.* 35:e00191. 10.1042/BSR20150025 25797907 PMC4413017

[B248] Van Der Wagen L. Te Boome L. Mansilla C. Lindemans C. Cuijpers M. Westinga K. (2014). Treatment of steroid resistant grade II to IV acute GVHD by infusion of mesenchymal stromal cells expanded with platelet lysate-a phase I/II study. *Cytotherapy* 16:S13. 10.1016/j.jcyt.2014.01.032 24321746

[B249] van Megen K. M. van’t Wout E.-J. T. Lages Motta J. Dekker B. Nikolic T. Roep B. O. (2019). Activated mesenchymal stromal cells process and present antigens regulating adaptive immunity. *Front. Immunol.* 10:694. 10.3389/fimmu.2019.00694 31001285 PMC6457321

[B250] Von Ahrens D. Bhagat T. D. Nagrath D. Maitra A. Verma A. (2017). The role of stromal cancer-associated fibroblasts in pancreatic cancer. *J. Hematol. Oncol.* 10:76. 10.1186/s13045-017-0448-5 28351381 PMC5371211

[B251] Vonk L. A. van Dooremalen S. F. J. Liv N. Klumperman J. Coffer P. J. Saris D. B. F. (2018). Mesenchymal stromal/stem cell-derived extracellular vesicles promote human cartilage regeneration in vitro. *Theranostics* 8 906–920. 10.7150/thno.20746 29463990 PMC5817101

[B252] Wang T. Sun S. Wan Z. Weil M. H. Tang W. (2012). Effects of bone marrow mesenchymal stem cells in a rat model of myocardial infarction. *Resuscitation* 83 1391–1396. 10.1016/J.RESUSCITATION.2012.02.033 22450658

[B253] Waterman R. S. Tomchuck S. L. Henkle S. L. Betancourt A. M. (2010). A new mesenchymal stem cell (MSC) paradigm: polarization into a pro-inflammatory msc1 or an immunosuppressive MSC2 phenotype. *PLoS One* 5:e10088. 10.1371/journal.pone.0010088 20436665 PMC2859930

[B254] Wei H.-J. J. Wu A. T. Hsu C.-H. H. Lin Y.-P. P. Cheng W.-F. F. Su C.-H. H. (2011). The development of a novel cancer immunotherapeutic platform using tumor-targeting mesenchymal stem cells and a protein vaccine. *Mol. Ther.* 19 2249–2257. 10.1038/mt.2011.152 21792181 PMC3242654

[B255] Wei X. Yang X. Han Z. Qu F. Shao L. Shi Y. (2013). Mesenchymal stem cells: a new trend for cell therapy. *Acta Pharmacol. Sin.* 34 747–754. 10.1038/aps.2013.50 23736003 PMC4002895

[B256] Wei X. X. Fong L. Small E. J. (2015). Prostate cancer immunotherapy with sipuleucel-t: current standards and future directions. *Expert. Rev. Vaccines* 14 1529–1541. 10.1586/14760584.2015.1099437 26488270

[B257] Wraith D. C. (2017). The future of immunotherapy: a 20-year perspective. *Front. Immunol.* 8:1668. 10.3389/fimmu.2017.01668 29234325 PMC5712390

[B258] Wu M. Zhang R. Zou Q. Chen Y. Zhou M. Li X. (2018). Comparison of the biological characteristics of mesenchymal stem cells derived from the human placenta and umbilical cord. *Sci. Rep.* 8:5014. 10.1038/s41598-018-23396-1 29568084 PMC5864926

[B259] Wu Y. Chen L. Scott P. G. Tredget E. E. (2007). Mesenchymal stem cells enhance wound healing through differentiation and angiogenesis. *Stem Cells* 25 2648–2659. 10.1634/stemcells.2007-0226 17615264

[B260] Xin H. Sun R. Kanehira M. Takahata T. Itoh J. Mizuguchi H. (2009). Intratracheal delivery of CX3CL1-expressing mesenchymal stem cells to multiple lung tumors. *Mol. Med.* 15 321–327. 10.2119/molmed.2009.00059 19603106 PMC2710294

[B261] Xu G. Zhang Y. Zhang L. Ren G. Shi Y. (2007). The role of IL-6 in inhibition of lymphocyte apoptosis by mesenchymal stem cells. *Biochem. Biophys. Res. Commun.* 361 745–750. 10.1016/j.bbrc.2007.07.052 17678624 PMC2699935

[B262] Yamada Y. Ueda M. Hibi H. Baba S. (2006). A novel approach to periodontal tissue regeneration with mesenchymal stem cells and platelet-rich plasma using tissue engineering technology: a clinical case report. *Int. J. Periodontics Restorative Dent.* 26 363–369. 16939018

[B263] Yorukoglu A. C. Kiter A. E. Akkaya S. Satiroglu-Tufan N. L. Tufan A. C. (2017). A concise review on the use of mesenchymal stem cells in cell sheet-based tissue engineering with special emphasis on bone tissue regeneration. *Stem Cells Int.* 2017 1–13. 10.1155/2017/2374161 29230248 PMC5694585

[B264] Yu T. T. L. Gupta P. Ronfard V. Vertès A. A. Bayon Y. (2018). Recent progress in European advanced therapy medicinal products and beyond. *Front. Bioeng. Biotechnol.* 6:130. 10.3389/fbioe.2018.00130 30298129 PMC6161540

[B265] Zeng X. Zeng Y.-S. Ma Y.-H. Lu L.-Y. Du B.-L. Zhang W. (2011). Bone marrow mesenchymal stem cells in a three-dimensional gelatin sponge scaffold attenuate inflammation, promote angiogenesis, and reduce cavity formation in experimental spinal cord injury. *Cell Transplant.* 20 1881–1899. 10.3727/096368911X566181 21396163

[B266] Zhang T. Lee Y. Rui Y. Cheng T. Jiang X. Li G. (2013). Bone marrow-derived mesenchymal stem cells promote growth and angiogenesis of breast and prostate tumors. *Stem Cell Res. Ther.* 4:70. 10.1186/scrt221 23763837 PMC3707041

[B267] Zhang Y. Ma L. Su Y. Su L. Lan X. Wu D. (2019). Hypoxia conditioning enhances neuroprotective effects of aged human bone marrow mesenchymal stem cell-derived conditioned medium against cerebral ischemia in vitro. *Brain Res.* 1725:14632. 10.1016/j.brainres.2019.146432 31491422

[B268] Zhu Y. Sun Z. Han Q. Liao L. Wang J. Bian C. (2009). Human mesenchymal stem cells inhibit cancer cell proliferation by secreting DKK-1. *Leukemia* 23 925–933. 10.1038/leu.2008.384 19148141

